# How Genomic and Structural Context Could Shape JAK-STAT Variant Pathogenicity

**DOI:** 10.1017/thg.2026.10054

**Published:** 2026-03-31

**Authors:** Markus Hoffmann, Hye Kyung Lee

**Affiliations:** 1 Department of Biochemistry and Molecular & Cellular Biology, Georgetown University Medical Center, Washington, DC, USA; 2 National Institute of Diabetes, Digestive and Kidney Diseases (NIDDK), US National Institutes of Health, Bethesda, MD, USA

**Keywords:** JAK-STAT pathway, Missense mutations, SNPs, Protein structure, Disease-associated variants, COSMIC, All of Us database, ClinVar

## Abstract

The Janus kinase (JAK)-Signal Transducer and Activator of Transcription (STAT) pathway is essential for cellular signal transduction, regulating immune responses, hematopoiesis, and cell proliferation. Dysregulation of JAK-STAT signaling due to genetic variations, particularly missense mutations, has been implicated in autoimmune disorders, cancers, and hematological malignancies. This study investigates missense mutations in JAK and STAT genes, focusing on disease-associated single nucleotide polymorphisms (SNPs) and ClinVar benign variants identified in the All of Us and COSMIC databases. We analyzed the distribution of these mutations across functional domains, their structural localization, and biochemical properties. We identified mutation hotspots within specific domains, highlighting their correlation with disease phenotypes. Structural mapping revealed that disease-associated SNPs predominantly localize in linker regions and at the boundaries of secondary structures, suggesting a significant impact on folding, stability, and function of JAK and STAT proteins. Additionally, we examined the genomic context of mutations and identified vulnerable sequences; for example, ‘GATC’. Furthermore, our analysis found no predominant association between potential CRISPR-Cas9 target sites and ClinVar benign/disease-associated SNPs. The analysis of amino acid sequence patterns surrounding mutations uncovered an enrichment of hydrophobic residues leucine (Leu), isoleucine (Ile), methionine (Met), and phenylalanine (Phe) in close proximity to disease-associated mutations. Our findings emphasize the importance of structural and biochemical context in determining pathogenicity. In this study, we provide a bioinformatic strategy for refining variant classification and understanding the roles of JAK-STAT pathway mutations in disease.

The Janus kinase (JAK)-Signal Transducer and Activator of Transcription (STAT) signaling pathway is crucial for gene regulation in common and lineage-specific genetic programs (Suppl. Fig. 1) (Brooks & Putoczki, 2020; X. Hu et al., 2021; Jankowski et al., 2021; Lee et al., 2021; Shillingford, 2002; Xue et al., 2023). Four known JAKs (JAK1, JAK2, JAK3, and TYK2) and seven STATs (STAT1, STAT2, STAT3, STAT4, STAT5A, STAT5B, and STAT6) collectively form the JAK and STAT gene families. The members of these gene families have crucial functions such as immune modulation, cell proliferation, and hematopoiesis (Brooks & Putoczki, 2020; X. Hu et al., 2021; Lee et al., 2021; Xue et al., 2023). The JAK and STAT genes carry mutations, such as single nucleotide polymorphisms (SNPs), representing the most prevalent form of genetic variation among individuals (L. X. Shen et al., 1999). These variations are characterized based on their genomic location and/or their potential influence on gene expression or function (Chu & Wei, 2019). In noncoding regions, such as promoters and enhancers, SNPs can play essential roles in gene regulation (Hecker et al., 2023; Hoffmann et al., 2023, 2025; Lee et al., 2018; Peña-Martínez & Rodríguez-Martínez, 2024). Within coding regions, SNPs are further classified into synonymous variants, which preserve the amino acid sequence and are functionally silent and evolutionarily neutral, and nonsynonymous (missense) variants, which change amino acids and can influence protein structure and/or function (Chu & Wei, 2019; Lio et al., 2025; Tsoy et al., 2024). It is well established that dysregulation of the JAK and STAT genes, through amino acid changes and altered regulatory element activity, can cause diverse pathophysiological outcomes such as autoimmune diseases, cancer, and infectious diseases (Erdogan et al., 2022; Hennighausen & Lee, 2020; Hoffmann, Willruth et al., 2024). Missense mutations drive functional changes of proteins by altering protein stability, disrupting protein-protein interactions, or compromising enzymatic functions, thus serving as potent drivers of disease (Teng et al., 2010).

Some mutations on JAK and STAT genes frequently lead to constitutive activation of the JAK-STAT pathway, a hallmark of various hematological malignancies. A well-known example is the JAK2^Val617Phe/V617F^ mutation, which is highly prevalent in myeloproliferative neoplasms, occurring in approximately 90–95% of polycythemia vera (PV) cases and 50–60% of individuals with essential thrombocythemia and primary myelofibrosis (Perner et al., 2019; Rampal et al., 2014). This gain-of-function mutation enhances JAK-STAT signaling even in the absence of cytokine stimulation, thereby driving uncontrolled cell proliferation and survival. Similarly, activating mutations in JAK1 and JAK3 have been identified in T-cell acute lymphoblastic leukemia (T-ALL), where they contribute to persistent JAK-STAT pathway activation (Girardi et al., 2018; Waldmann, 2017). Beyond hematological malignancies, dysregulation of the JAK-STAT pathway has been implicated in solid tumors and autoimmune diseases, underscoring its broader significance in human pathology (Łączak et al., 2022; O’Shea et al., 2015). Mutations in STAT3, for instance, are linked to increased tumor invasiveness and poor clinical outcomes across multiple cancer types (Deng et al., 2022; Klein et al., 2021).

In our previous work (Hoffmann & Hennighausen, 2025), we conducted a large-scale survey of missense mutations within the JAK and STAT genes across two major repositories — the All of Us database (The All of Us Research Program Genomics Investigators et al., 2024), which captures a broad range of genetic variation from the general but mostly healthy population in the United States, and the COSMIC (Bamford et al., 2004; Sondka et al., 2024) database, a leading resource for somatic mutations in cancer. Our investigation identified hundreds of unique amino acid–changing variants, some of which had been reported as disease-associated. This analysis provided a resource cataloging the breadth and frequency of missense mutations in JAK-STAT genes. Building on these findings, this study aims to focus explicitly on the identified disease-causing (by literature) SNPs and ClinVar (Landrum et al., 2014) benign/likely benign SNPs and their distribution among specific functional domains of JAK and STAT proteins. We assess whether certain functional domains are more frequently affected and how these mutations correlate with disease. By mapping the structural distribution of disease-associated SNPs, we determine whether they predominantly occur on the protein surface or within its core. Additionally, we analyze specific target sequences, CRISPR target proximity, and amino acid composition patterns to identify shared vulnerabilities between disease-causing and ClinVar benign SNPs that may explain why specific regions are more mutation- or disease-prone.

## Materials and Methods

### All of Us Data Explorer

The All of Us Research Program gathers health and genomic data from participants residing in the U.S. We accessed the All of Us Controlled Tier Dataset v7 (All of Us Research Program Genomics Investigators et al., 2024) (encompassing 413,000 participants) through the Data Browser to examine SNPs. We focused on missense mutations in the JAK-STAT gene families, specifically SNPs that alter amino acids, and we analyzed their frequency across various demographic groups. All data were anonymized according to program protocols. In accordance with All of Us guidelines, we included only missense mutations (excluding all other SNP types) identified in at least 20 participants and previously associated with a disease in the literature. The cut-off of 20 is dictated by the policy of All of Us due to privacy concerns. We focused only on missense mutations because they change one amino acid to another and therefore could have a significant impact on protein function (Pal & Moult, 2015).

### COSMIC (Catalogue of Somatic Mutations in Cancer)

The Catalogue of Somatic Mutations in Cancer (COSMIC) database (https://cancer.sanger.ac.uk/cosmic; Bamford et al., 2004; Sondka et al., 2024) tracks somatic mutations identified in cancer. Using COSMIC v100 (>1,000,000 tumor samples), we examined missense mutations in the JAK-STAT pathway, focusing on those found in cancer samples. COSMIC details the mutational spectrum, tissue distribution, and associated cancers for each SNP, enabling direct comparison with findings from the All of Us cohort. We extracted mutations using COSMIC’s online tools, filtering by mutation type. For each SNP, we noted the number of samples carrying the mutation and referenced disease associations from existing literature. To align with the All of Us approach, we included only missense mutations (excluding all other SNP types) present in at least 20 tumor samples and that were previously associated with a disease in the literature. We used the cut-off of 20 as dictated by the policy of All of Us due to privacy concerns to have a consistent filter between the two databases.

### Obtaining SNPs That are Disease-Associated in the Published Literature and SNPs Classified as Benign in ClinVar

**Figure 1.**
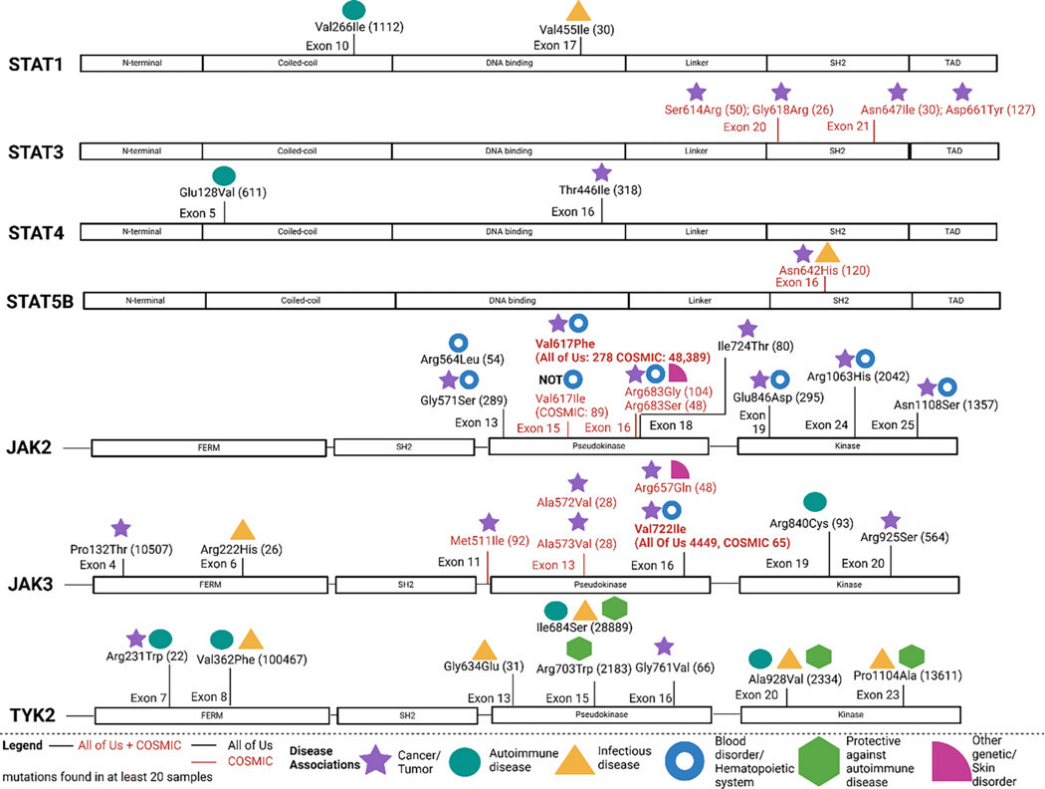
Domain-specific distribution of missense mutations in JAK and STAT proteins and their associations with disease. The schematic representation of JAK (JAK2, JAK3, TYK2) and STAT (STAT1, STAT3, STAT4, STAT5B) proteins highlights the locations of missense mutations identified in the All of Us and COSMIC databases. Symbols indicate disease associations, including autoimmune diseases (turquoise circles), cancer/tumor (purple stars), infectious diseases (yellow triangles), blood disorders/hematopoietic system involvement (blue donuts), protective mutations against autoimmunity (green hexagons), and other genetic disorder order skin disorder (dark pink quarter of a circle). Mutations found in at least 20 individuals are labeled, with mutations found in All of Us (black font) or COSMIC (red font) and mutations found in All of Us and COSMIC are highlighted in bold red. This visualization provides insight into mutation clustering within functional domains. Protein domains are annotated as follows: STAT proteins include the N-terminal, coiled-coil, DNA-binding, linker, Src homology 2 (SH2), and transactivation (TAD) domains, while JAK proteins include the FERM (For protein 4.1, Ezrin, Radixin, and Moesin), SH2, pseudokinase, and kinase domains.

We used the identified SNPs and literature from Hoffmann and Hennighausen (2025). We used the Athena – OHDSI Vocabularies Repository database (https://athena.ohdsi.org/search-terms/start) to generalize disease terms (Figure 1, Suppl. Table 1) (Reich et al., 2024).

We used the NCBI Entrez E-utilities API (https://eutils.ncbi.nlm.nih.gov/entrez/eutils/esearch.fcgi, with parameter “db”: “clinvar”) to filter all missense mutations from All of Us and COSMIC for only mutations categorized as benign by ClinVar.

The Python code can be found at GitHub: https://github.com/Firestar93/JAKSTAT_missenseSNPs_properties

### Enzyme Cut Sites and CRISPR Sites on the Nucleotide Sequence and Amino Acid Composition Analysis on the Amino Acid Sequences

We downloaded the mRNA sequences from the CCDS database (Farrell et al., 2014; Harte et al., 2012; Pruitt et al., 2009; Pujar et al., 2018) for the following genes: *STAT1* (CCDS2309.1), *STAT3* (CCDS32656.1), *STAT4* (CCDS2310.1), *STAT5B* (CCDS11423.1), *JAK*2 (CCDS6457.1), *JAK3* (CCDS12366.1), and *TYK2* (CCDS12236.1).

We used a house-made Python script to detect enzyme cut sites (using the Biopython package, specifically the Bio. Restriction module (Cock et al., 2009)) and the following CRISPR-Cas sites: SpCas9 recognizes the 5“-NGG-3” sequence, while SaCas9 targets 5“-NNGRRT-3”. NmCas9 requires the 5“-NNNNGATT-3” PAM, and St1Cas9 recognizes 5“-NNAGAAW-3”. Similarly, St3Cas9 targets 5“-NGGNG-3”, and CjCas9 recognizes the 5“-NNNNACA-3” sequence. FnCas9 operates with a 5“-YG-3” PAM, whereas TdCas9 exhibits specificity for 5“-NAAAW-3”. SpCas9-NG (SpG) has a relaxed PAM requirement of 5“-NG-3”, broadening its targeting scope. Lastly, SpRY functions as a near-PAM-less variant, with a preference for NRN sequences. To associate enzyme sites and CRISPR sites to a mutation, we stretch a window of 20 bps in each direction of the mutation since it was reported that 20 bps ensures that any potential restriction sites are adequately captured (Wang, 2018). We used the UpSet Python library for visualizations (Lex et al., 2014).

We used house-made Python scripts to detect amino acid combinations close (3 amino acids upstream and downstream) to ClinVar benign and disease-associated mutations using the Biopython package.

The code can be found at GitHub: https://github.com/Firestar93/JAKSTAT_missenseSNPs_properties

### Visualization of the Protein Structures

We used the ChimeraX tool for the visualization of the protein structures (Goddard et al., 2018; Meng et al., 2023; Ucsf Chimerax Pettersen et al., 2021). We used ALPHAFOLD MONOMER V2.0 (Jumper et al., 2021) predicted structures for the following proteins: STAT1 (AF-P42224-F1-model_v4), STAT3 (AF-P40763-F1-model_v4), STAT4 (AF-Q14765-F1-model_v4), STAT5B (AF-P51692-F1-model_v4), JAK2 (AF-O60674-F1-model_v4), JAK3 (AF-P52333-F1-model_v4), and TYK2 (AF-P29597-F1-model_v4). ChimeraX files for interactive visualization can be found at figshare: https://doi.org/10.6084/m9.figshare.28597121.

## Results

To better understand the characteristics of JAK and STAT mutations in terms of their location within the nucleotide and amino acid sequence, we focused on their distribution across protein domains and disease associations for STAT1, STAT3, STAT4, STAT5B, JAK2, JAK3, and TYK2. We could not identify any missense variants in STAT2, STAT5A, STAT6, and JAK1 that satisfied the All of Us requirements of at least 20 individuals harboring them. We identified domains particularly susceptible to disease-related alterations. We further assessed where they are located to evaluate their effects on protein structure and stability. Beyond structural localization, we analyzed the genomic context of these mutations, examining their proximity to specific feasible sequences and CRISPR target sites to assess potential regulatory influences and editing feasibility. Finally, we investigated conserved amino acid sequence patterns near disease-associated mutations to identify motifs contributing to mutation susceptibility.

### Mutation Hotspots in JAK-STAT Proteins and Their Disease Relevance

We visualized mutations that were disease-associated with autoimmune disease, cancer, chronic disease, etc. (Figure 1, Suppl. Table 1) and ClinVar benign (Suppl. Fig. 2) from the All of Us and COSMIC database and investigated distinct patterns in the distribution of disease-associated SNPs across JAK and STAT proteins to highlight specific domains linked to various diseases. Autoimmune disorders were mainly associated with mutations in the coiled-coil domains of STAT1 (Uzel et al., 2013) and STAT4 (Saevarsdottir et al., 2022) critical for dimerization and transcriptional activity. In contrast, cancer-related mutations were predominantly found in the SH2 domains of STAT3 (Cheon et al., 2022; D. Kim et al., 2021; Kristensen et al., 2014; Olson et al., 2020; Ramsey et al., 2023; Rivero et al., 2021; M. Shen, 2023; Yan et al., 2015) and STAT5B (Freiche et al., 2022; Z. Hu et al., 2023; Yin et al., 2023), suggesting that alterations in these key signaling interfaces contribute to tumorigenesis. Hematological malignancies and blood disorders are strongly associated with SNPs in the Pseudokinase (Arai et al., 2023; Delio et al., 2023; Eichstaedt et al., 2020; Haji Paiman et al., 2024; Krah et al., 2023; Panovska-Stavridis et al., 2016) and kinase (Kapralova et al., 2016; Maaziz et al., 2023; Tun et al., 2022) domains of JAK2, regions essential for modulating JAK-STAT signaling. We can also observe a plethora of literature associating the JAK2 Pseudokinase (Bahar et al., 2016; Bourrienne et al., 2024; Carreño-Tarragona et al., 2021; Choi et al., 2024; Gupta, Varma, Kumar et al., 2023; Gupta, Varma, Sreedharanunni et al., 2023; Hassan et al., 2022; Krah et al., 2023; Lin et al., 2019; Mambet et al., 2018; Pace et al., 2023; Patchell et al., 2024; Puli’uvea et al., 2024; Roncero et al., 2016; Schulze et al., 2019; Skoczen et al., 2020; Veitia & Innan, 2022; R. Z. Xu et al., 2022; Yongchao Zhang et al., 2024) and kinase (Benton et al., 2019; Kapralova et al., 2016; Mambet et al., 2018; Schulze et al., 2019) domains to cancer. Similarly, cancer-related mutations were frequently observed in the Pseudokinase domain of JAK3 (Agarwal et al., 2015; Bergmann et al., 2014; Bouchekioua et al., 2014; de Martino et al., 2013; Ehrentraut et al., 2016; Koo et al., 2012; Rivera-Munoz et al., 2018; Sato et al., 2008; Sim et al., 2017; L. Xu et al., 2016), reinforcing its role in malignant transformation. Infectious diseases, on the other hand, appeared to be linked to mutations in the FERM domain of JAK3 (Zhong et al., 2017), the DNA binding domain of STAT1 (Uzel et al., 2013), and all over TYK2 (Kerner et al., 2021, 2019; Ogishi et al., 2022), which play a crucial role in cytokine receptor binding and immune response regulation. Interestingly, mutations were associated with autoimmunity (Li et al., 2013; López-Isac et al., 2016; Motegi et al., 2019) and protective against autoimmunity (Diogo et al., 2015; Enerbäck et al., 2018; Jensen et al., 2023; Motegi et al., 2019) in TYK2, suggesting that variations in these regions may provide resilience against autoimmune diseases, which is an ongoing field of study (Molitor et al., 2025; Syed et al., 2025).

### Examining the Relationship Between Structural Alterations in JAK-STAT Proteins and the Pathogenic Potential of Mutations

**Figure 2.**
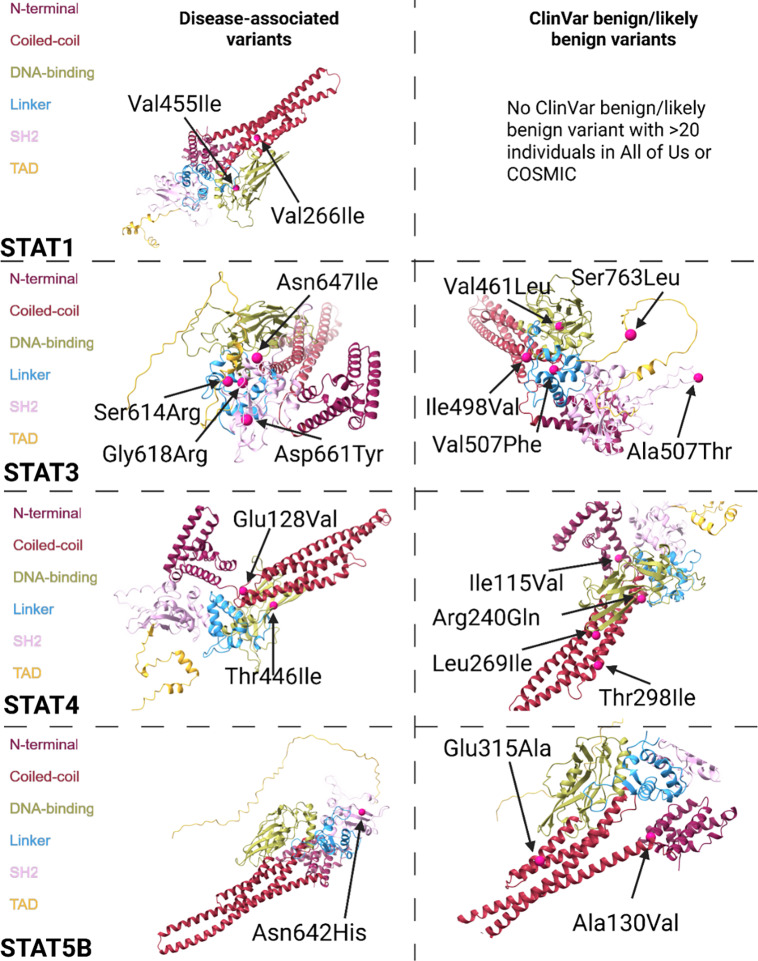
Structural analysis of disease-associated and ClinVar benign missense variants in the STAT proteins. The panel illustrates the secondary structure localization of disease-associated (left) and benign/likely benign (right) mutations mapped onto AlphaFold predicted protein structures.

**Figure 3.**
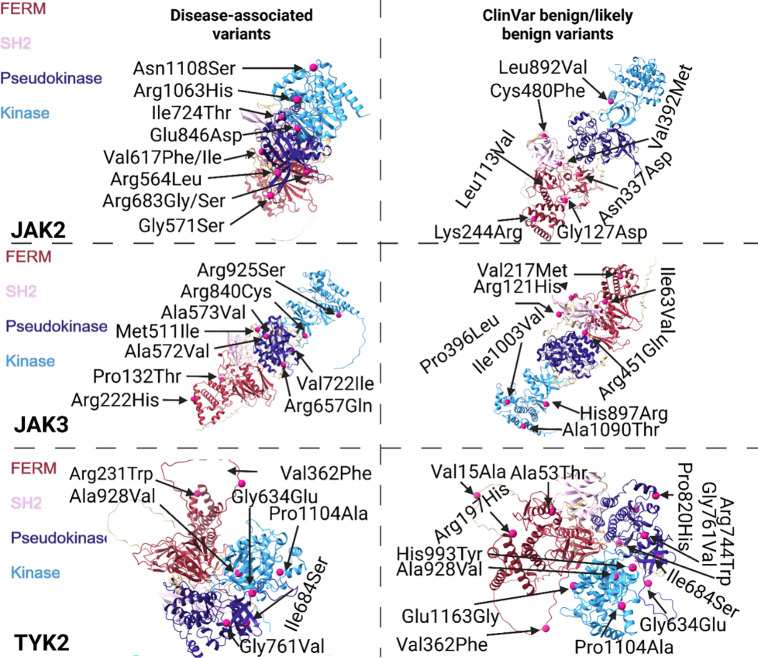
Structural analysis of disease-associated and ClinVar benign missense variants in the JAK proteins. The panel illustrates the secondary structure localization of disease-associated (left) and benign/likely benign (right) mutations mapped onto AlphaFold predicted protein structures.

Next, we investigated the protein structure using the AlphaFold3 AI-predicted model to determine where the mutations impact structural alterations. We observed that disease-associated SNPs were more frequently found in linker regions connecting secondary structural elements, such as between alpha helices and beta sheets. When these mutations occurred within an alpha helix or beta sheet, they were predominantly located at the boundary of the structure, with only rare occurrences in the middle (Figure 2, Figure 3). This suggests that mutations in transition regions may have a more significant impact on protein dynamics and function, potentially altering folding, stability, or interactions with other molecules. In contrast, benign mutations were more often embedded within well-defined secondary structures, but rarely in linker regions. Furthermore, we observed that disease-associated mutations were predominantly located within the interior of the protein’s 3D structure, suggesting that these variants may impact structural integrity or disrupt key protein-protein interactions. Conversely, benign mutations were more commonly found on surface-exposed regions of the protein (i.e., an amino acid that faces the outside of the protein) (Suppl. Fig. 3).

### Conserved Amino Acid Patterns in Proximity to Disease-Associated and ClinVar Benign Variants

**Figure 4.**
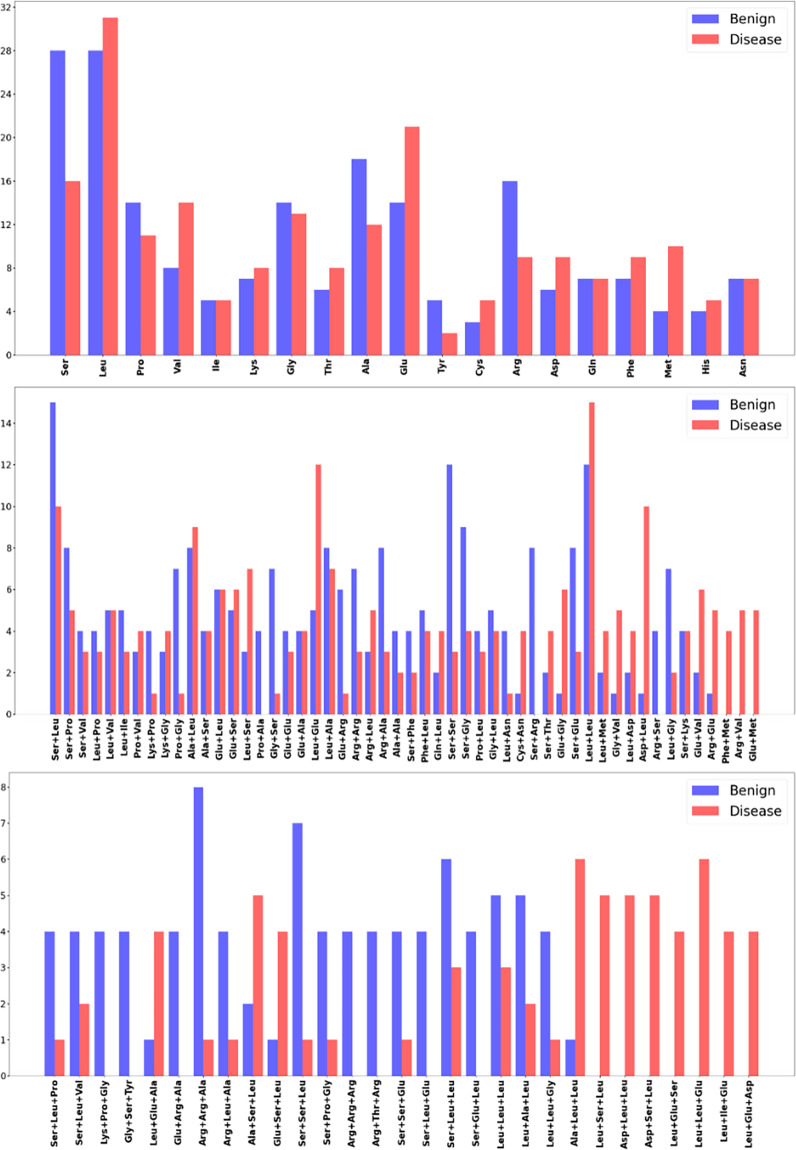
Comparative analysis of amino acid patterns (one amino acid, two amino acid combinations, and three amino acid combinations out of three upstream and three downstream of the variant in All of Us or COSMIC) in benign and disease-associated variants. Amino acid compositions for benign variants are blue, and disease variants are red.

We further compared amino acid patterns surrounding the ClinVar benign and disease-associated mutations (three amino acids upstream and three amino acids downstream of the mutated site (see Materials and Methods, Figure 4). This comparative analysis of amino acid patterns in benign and disease-associated variants revealed distinct compositional differences across disease-associated and ClinVar benign variants. In the single-residue analysis (top panel), certain amino acids, such as valine (Val), glutamic acid (Glu), and methionine (Met), exhibited higher frequencies in proximity to disease-associated variants compared to benign variants, while others, such as serine (Ser), alanine (Ala), tyrosine (Tyr), and arginine (Arg), appeared more frequently in benign variants.

At the dipeptide level (second panel), several combinations were more prominent in proximity to disease-associated mutations such as (1) leucine and glutamic acid (Leu+Glu), (2) leucine and leucine (Leu+Leu), (3) aspartic acid and leucine (Asp, Leu), and (4) arginine and glutamic acid (Arg+Glu). Some combinations were uniquely present close to disease-associated variants: (1) phenylalanine and methionine (Phe+Met), (2) arginine and valine (Arg+Val), and (3) glutamic acid and methionine (Glu+Met).

Expanding to combinations of three amino acids out of six surrounding amino acids (third panel), we observed some combinations that are uniquely in proximity to disease-associated variants: (1) leucine, serine, leucine (Leu+Ser+Leu), (2) aspartic acid, leucine, leucine (Asp+Leu+Leu), (3) aspartic acid, serine, leucine (Asp, Ser, Leu), (4) leucine, leucine, glutamic acid (Leu+Leu+Glu), (5) leucine, isoleucine, glutamic acid (Leu+Ile+Glu), and (6) leucine, glutamic acid, aspartic acid (Leu+Glu+Asp). On the other hand, we detected the following combinations only close to benign variants (1) lysine, proline, glycine (Lys+Pro+Gly), (2) glycine, serine, tyrosine (Gly+Ser+Tyr), (3) arginine, arginine, arginine (Arg+Arg+Arg), and (4) arginine, threonine, arginine (Arg+Thr+Arg).

The observed differences in amino acid compositions surrounding disease-associated and benign variants suggest underlying structural and functional constraints that contribute to pathogenicity. A key trend is the enrichment of hydrophobic residues, particularly leucine (Leu), isoleucine (Ile), methionine (Met), and phenylalanine (Phe), in proximity to disease-associated variants. The frequent occurrence of Leu+Leu, Leu+Glu, and Leu+Glu+Asp combinations suggests that these mutations often occur within hydrophobic cores, where they may disrupt protein stability or alter packing interactions. Similarly, the presence of methionine (Met) in disease-associated motifs, such as Glu+Met and Phe+Met, points to potential disruptions in hydrophobic regions, particularly in proteins involved in enzymatic activity or membrane function. Conversely, benign variants appear to favor polar and flexible residues, with an overrepresentation of serine (Ser), glycine (Gly), and threonine (Thr). The exclusive presence of Gly+Ser+Tyr and Lys+Pro+Gly in benign variants suggests that these substitutions predominantly occur in solvent-exposed loops or linker regions, where flexibility and structural adaptability mitigate the effects of mutation. Additionally, the recurrent occurrence of arginine-rich motifs (Arg+Arg+Arg and Arg+Thr+Arg) in benign variants indicates that these substitutions are likely involved in electrostatic interactions or protein-protein binding sites that can accommodate mutational changes without significant functional consequences. A second striking pattern is the enrichment of negatively charged residues (glutamic acid, Glu, and aspartic acid, Asp) near disease-associated variants, suggesting potential disruptions in salt-bridge interactions and protein stability. The presence of Arg+Glu, Asp+Leu+Leu, and Leu+Glu+Asp in disease-associated variants indicates that these mutations may destabilize electrostatic interactions or affect protein folding. In contrast, the benign variants tend to retain positively charged arginine (Arg), which is commonly involved in stabilizing protein structures or mediating protein-protein interactions. Taken together, these findings suggest that disease-associated variants frequently occur in structurally constrained regions, where mutations disrupt core hydrophobic interactions, electrostatic balance, or functional interfaces. In contrast, benign variants are more likely to appear in flexible or surface-exposed regions, where mutations are better tolerated due to the preservation of local structural dynamics. The distinct differences in amino acid preferences between disease and benign variants provide insights into the physicochemical constraints that contribute to pathogenicity and may aid in refining predictive models for variant classification.

### Analyzing the Nucleotide Sequence Near Benign and Disease-Associated Variants

**Figure 5.**
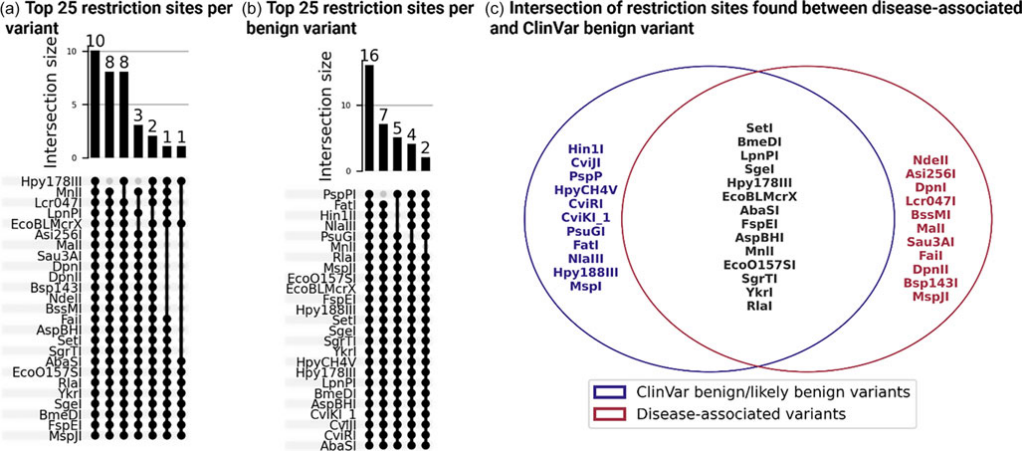
Enzyme restriction site analysis in proximity to disease-associated and ClinVar benign variants in JAK and STAT genes. (a,b) The top 25 restriction enzymes identified near disease-associated and benign variants, respectively. (c) Venn diagram illustrating the overlap of restriction sites found near disease-associated variants (red) and ClinVar benign variants (blue).

To explore the genomic context of disease-associated and ClinVar benign mutations, we analyzed the nucleotide sequences surrounding these variants, focusing on a 20 bp region around each mutation. The first part of our investigation aimed to identify patterns of nucleotide sequences and assess whether specific sequences are preferentially found near disease-associated or benign mutations (Figure 5, Suppl. Fig. 4). Our analysis identified nucleotide sequences of 625 restriction enzyme sites out of a total of 1,088 within the examined regions. Notably, several nucleotide sequences were exclusively present near disease-associated mutations but absent in ClinVar benign variants. These included the nucleotide sequences of DpnI, Asi256I, DpnII, MalI, Lcr047I, NdeII, Bsp143I, Sau3AI, FaiI, MspJI, and BssMI. We found that the nucleotide sequence ‘GATC’ was predominantly present near disease-associated mutations but was entirely absent in the vicinity of ClinVar benign mutations.

**Figure 6.**
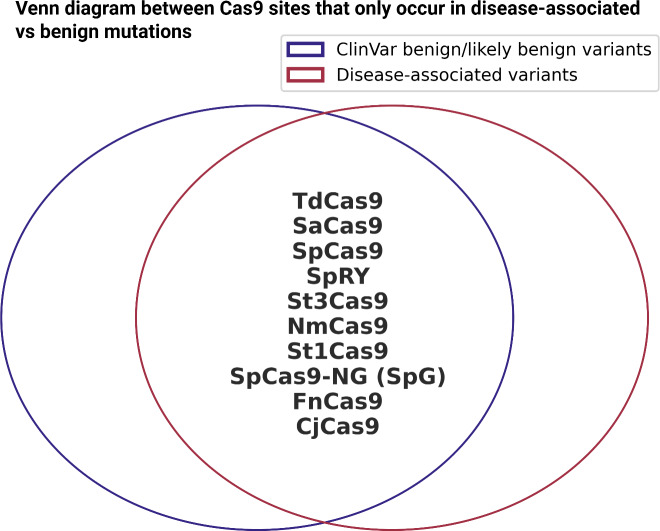
CRISPR cut site analysis in proximity to disease-associated and benign mutations. Venn diagram illustrating the overlap of Cas9 cut sites uniquely occurring in either disease-associated (red) or benign (blue) mutations.

The second part of this analysis is to assess whether multiple Cas9 enzymes preferentially target sequences near disease-associated or ClinVar benign mutations; we analyzed the presence of Cas9 recognition sites within a 20 bp region surrounding these mutations (Figure 6, Suppl. Fig. 5). We examined a range of Cas9 enzymes, including SpCas9, SaCas9, NmCas9, St1Cas9, St3Cas9, CjCas9, FnCas9, TdCas9, xCas9, SpCas9-NG (SpG), SpRY, HiFi Cas9 (HF1), eSpCas9 (1.1), and HypaCas9 that recognized different PAM sequences (Suppl. Table 2) (Acharya et al., 2024; Du et al., 2023; Guo et al., 2019; Hibshman et al., 2024; Hou et al., 2013; Ikeda et al., 2019; H. K. Kim et al., 2020; Liang et al., 2022; Müller et al., 2016; Schmidheini et al., 2024; Slaymaker et al., 2016; Vakulskas et al., 2018; Wu et al., 2020; Yifei Zhang et al., 2020). Our analysis did not reveal a strong preference for any Cas9 protein’s cut site being predominantly located near disease-associated mutations, but absent in ClinVar benign mutations. These findings suggest that while CRISPR/Cas9 target sites are present around the variants, there is no clear enrichment in disease-associated sites that would indicate preferential targetability.

## Discussion

This study provides a comprehensive analysis of missense mutations in the JAK-STAT pathway, highlighting differences between disease-associated and ClinVar benign variants regarding structural localization, biochemical properties, and genomic context. By integrating mutation data from the All of Us and COSMIC databases, we identified properties that may help explain why specific variants contribute to disease while others remain functionally neutral. Our findings suggest that disease-associated mutations frequently disrupt core hydrophobic interactions, electrostatic balance, or functional interfaces, whereas benign variants are more commonly found within secondary structures like helices and sheets and also in surface-exposed regions, where structural constraints are less restrictive. These observations reinforce the notion that pathogenicity is not merely a function of amino acid substitution alone but is heavily influenced by protein architecture and nearby context on the nucleotide and amino acid level.

Our current understanding of disease-associated mutations is largely based on the analysis of individual SNPs, whereas real-world genetic variation often involves combinations of mutations that may interact in yet unknown ways. The lack of experimental data on epistatic interactions is a major limitation (Suppl. Text 1: Limitations and considerations) in mutation interpretation, as the functional impact of a given variant may depend on the presence of additional mutations within the same gene or pathway (Blumenthal et al., 2020; Hernández-Lorenzo et al., 2022; Hoffmann, Poschenrieder et al., 2024). While our stringent inclusion criteria (minimum 20 occurrences due to All of Us policy) allowed for robust analysis of more prevalent variants, it is important to acknowledge that rare yet highly pathogenic mutations, especially those associated with rare inherited disorders, might not be captured by this approach.

The GATC sequence is well known in bacteria as a recognition site for DNA adenine methyltransferase (Dam) and plays key roles in DNA repair, replication timing, and gene regulation (Flusberg et al., 2010). In *E. coli*, for example, GATC sites guide mismatch repair machinery to correct errors on the newly synthesized DNA strand (Horton et al., 2015). Although human cells do not use the same bacterial repair system, recent work shows that sequence context, including short motifs like GATC, can influence where replication errors occur and how repair systems handle them (Hasenauer et al., 2025). GATC-like sequences can also be recognized by certain transcription factors or occur in open chromatin regions, meaning changes nearby could alter gene expression (Mardenborough et al., 2019).

Future work could explore whether selected missense variants influence STAT dimer formation by combining state-aware structural hypotheses with independent computational estimates of interface perturbation and focused experimental validation, recognizing that dimerization is conformation-dependent and not fully captured by static structural predictions alone.

Understanding genetic variation requires considering not only individual mutations but also their broader structural and functional context. As data and experimental tools improve, distinguishing ClinVar benign from disease-associated variants will become more precise. Addressing gaps such as epistatic interactions and underreported mutations will clarify how JAK-STAT variants affect health. Integrating computational models of protein–protein interactions with high-throughput experiments could systematically investigate multi-variant and epistatic effects. In vivo studies introducing predicted single or combined SNPs into homozygous mouse lines, then crossbreeding and applying RNA-seq, ChIP-seq, and allergenic challenge assays (Gad, 1994), could reveal how complex mutation patterns shape pathway function. Clinical sample analysis will be critical to assess whether variants labeled as benign may contribute to disease in certain contexts, with our curated references offering a starting point for prioritization. Examining noncoding regulatory regions for pathogenic mutations may uncover additional mechanisms, but current datasets (All of Us, COSMIC) lack matched transcriptomic or epigenomic data, and patient samples were unavailable for this study. Future integration of genomic variation with matched expression and chromatin profiles will be essential for linking regulatory mutations to function. Combining large-scale genomics with advanced experimental systems offers a path to further investigate JAK-STAT variation and translate these insights into future therapeutic advances.

## Supporting information

Hoffmann and Lee supplementary material 1 Hoffmann and Lee supplementary material

Hoffmann and Lee supplementary material 2 Hoffmann and Lee supplementary material

## Data Availability

This study used data from the All of Us Research Program’s controlled Tier Dataset v.7, available to authorized users on the Researcher Workbench (https://databrowser.researchallofus.org/). Data from COSMIC v100 is available at (https://cancer.sanger.ac.uk/cosmic). The Python code can be found at: https://github.com/Firestar93/JAKSTAT_missenseSNPs_properties. Intermediate results and files can be found at: https://doi.org/10.6084/m9.figshare.28597121.

## Supplementary material

To view supplementary material for this article, please visit https://doi.org/10.1017/thg.2026.10054.
