# Supplementary material for: How Genomic and Structural Context Could Shape JAK-STAT Variant Pathogenicity
Source: Twin Res Hum Genet. 2026 Mar 31:1–13. Online ahead of print. doi: 10.1017/thg.2026.10054 (PMC13107192; doi:10.1017/thg.2026.10054)
Supplement: Hoffmann and Lee supplementary material 1 — Hoffmann and Lee supplementary material [file S1832427426100541sup001.docx]

*Supplementary Materials for:*

**How genomic and structural context could shape JAK-STAT variant pathogenicity**

Markus Hoffmann^1,2,*^ and Hye Kyung Lee^2,*^

^1^ Department of Biochemistry and Molecular & Cellular Biology, Georgetown University Medical Center, Washington, D.C., 20007, USA

^2^ Laboratory of Genetics and Physiology, National Institute of Diabetes, Digestive, and Kidney Diseases (NIDDK), US National Institutes of Health (NIH), Bethesda, Maryland, 20892, USA

*Corresponding authors: [mh2437@georgetown.edu](mailto:mh2437@georgetown.edu) ; [hyekyung.lee@nih.gov](mailto:hyekyung.lee@nih.gov)

**ABSTRACT**

**Background:** The Janus kinase (JAK)-Signal Transducer and Activator of Transcription (STAT) pathway is essential for cellular signal transduction, regulating immune responses, hematopoiesis, and cell proliferation. Dysregulation of JAK-STAT signaling due to genetic variations, particularly missense mutations, has been implicated in autoimmune disorders, cancers, and hematological malignancies. **Methods:** This study investigates missense mutations in JAK and STAT genes, focusing on disease-associated single nucleotide polymorphisms (SNPs) and ClinVar benign variants identified in the All of Us and COSMIC databases. We analyzed the distribution of these mutations across functional domains, their structural localization, and biochemical properties. **Results:** We identified mutation hotspots within specific domains, highlighting their correlation with disease phenotypes. Structural mapping revealed that disease-associated SNPs predominantly localize in linker regions and at the boundaries of secondary structures, suggesting a significant impact on folding, stability, and function of JAK and STAT proteins. Additionally, we examined the genomic context of mutations and identified vulnerable sequences, e.g., “GATC”. Furthermore, our analysis found no predominant association between potential CRISPR-Cas9 target sites and ClinVar benign/disease-associated SNPs. The analysis of amino acid sequence patterns surrounding mutations uncovered an enrichment of hydrophobic residues (leucine (Leu), isoleucine (Ile), methionine (Met), phenylalanine (Phe)) in close proximity to disease-associated mutations. **Conclusion:** Our findings emphasize the importance of structural and biochemical context in determining pathogenicity. With this study, we provide a bioinformatic strategy for refining variant classification and understanding the roles of JAK-STAT pathway mutations in disease.

Contents

[Supplementary Figure 1: The JAK-STAT pathway 2](#_Toc221103258)

[Supplementary Figure 2: SNPs in All of Us and COSMIC that are characterized benign in ClinVar 3](#_Toc221103259)

[Supplementary Figure 3: Amino acids that face the surface of the protein that were mutated and classified as disease-associated or ClinVar benign/likely benign. 4](#_Toc221103260)

[Supplementary Figure 4: Analysis of the sequence in 20bp proximity around disease-associated and ClinVar benign missense variant found in the All of Us and COSMIC database in terms of enzyme restriction sites 5](#_Toc221103261)

[Supplementary Figure 5: Analysis of the sequence in 20bp proximity around disease-associated and ClinVar benign missense variant found in the All of Us and COSMIC database in terms of CRISPR cut sites 6](#_Toc221103262)

[Supplementary Table 1: Amino acid changing SNPs in the STAT gene family 7](#_Toc221103263)

[Supplementary Table 2: CRISPR-Cas9 PAM sequences for the different Cas9 variants. 9](#_Toc221103264)

[Supplementary Text 1: Limitations and considerations 10](#_Toc221103265)

[REFERENCES 12](#_Toc221103266)

Supplementary Figure 1: The JAK-STAT pathway


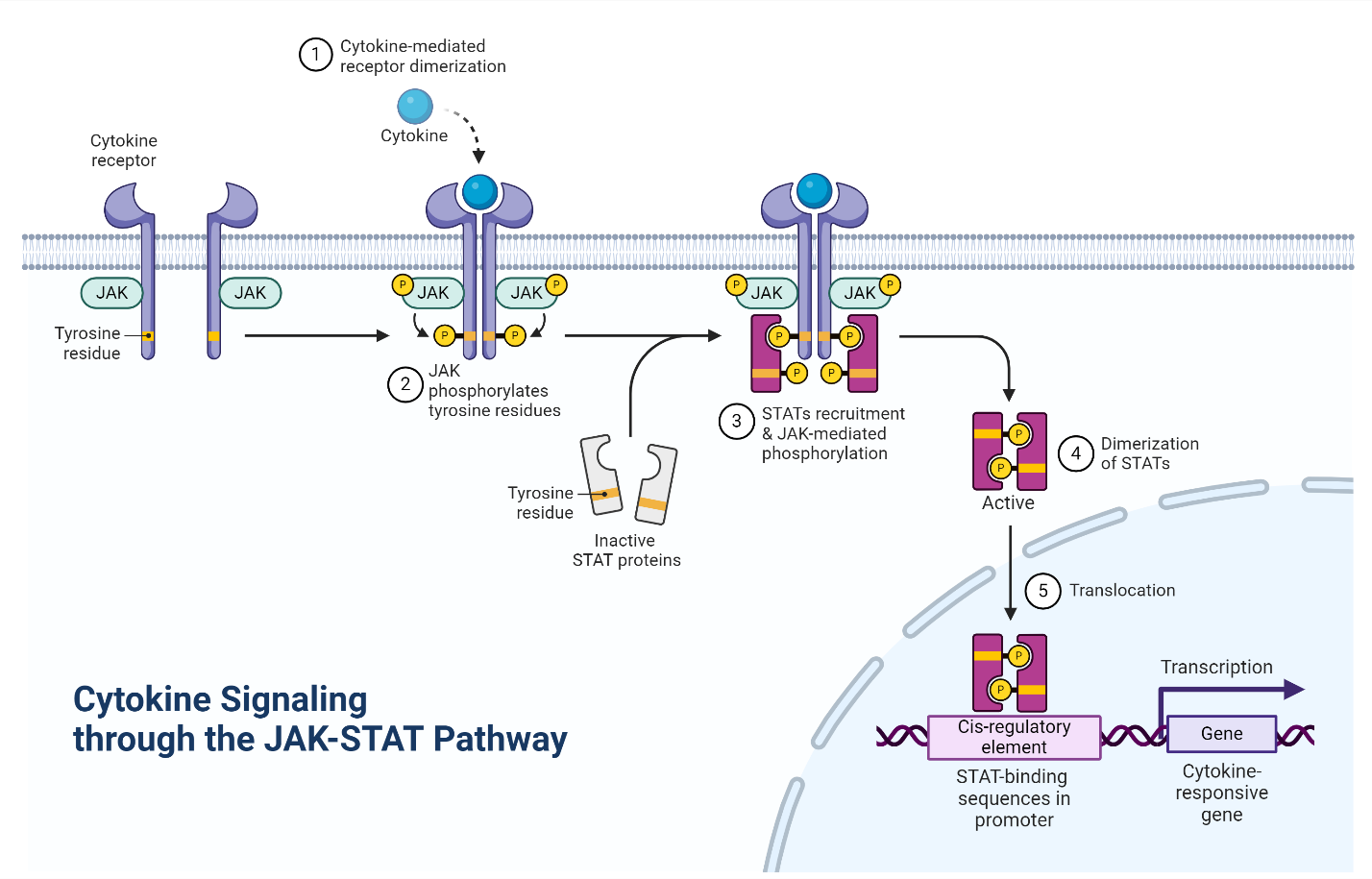


**Supplementary Figure 1:** The activation of the JAK-STAT pathway is critical in the immune system [1], leading to the regulation of immune genes, which play a significant role in the body's response to viral infections [2,3], autoimmune diseases [4], cancer [5], and a plethora of other conditions [6,7]. Following cytokine signaling, the JAK-STAT pathway facilitates the phosphorylation and dimerization of STAT transcription factors (TFs), which subsequently translocate to the nucleus to bind gamma-activated sites (GAS) motifs in cis-regulatory elements (CREs) such as promoters and enhancers [1] and regulate various immune genes (Figure 1 a, [8]). The successful and targeted binding of the STAT TF family with GAS motifs is crucial in regulating the expression levels of immune genes.

# Supplementary Figure 2: SNPs in All of Us and COSMIC that are characterized benign in ClinVar


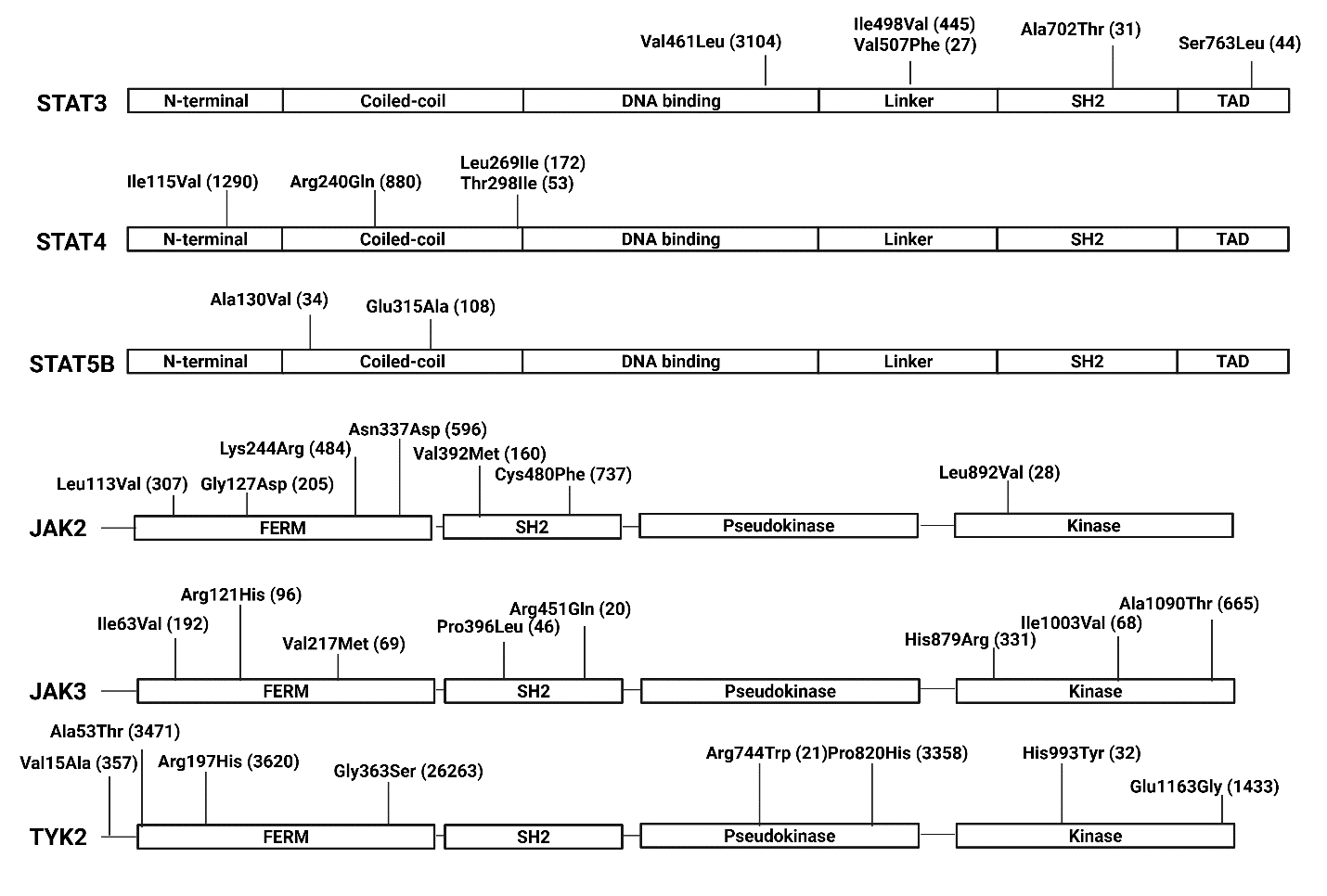


**Supplementary Figure 2:** Domain-specific distribution of ClinVar-classified benign SNPs in JAK and STAT proteins. The schematic representation highlights the locations of benign missense mutations across JAK (JAK2, JAK3, TYK2) and STAT (STAT3, STAT4, STAT5B) proteins. Mutations are mapped to key functional domains, including coiled-coil, SH2, FERM, Pseudokinase, and kinase regions, providing insight into their structural localization. Exon numbers are indicated for reference. This dataset serves as a comparative framework for understanding the structural contexts of benign versus disease-associated mutations.

# Supplementary Figure 3: Amino acids that face the surface of the protein that were mutated and classified as disease-associated or ClinVar benign/likely benign.


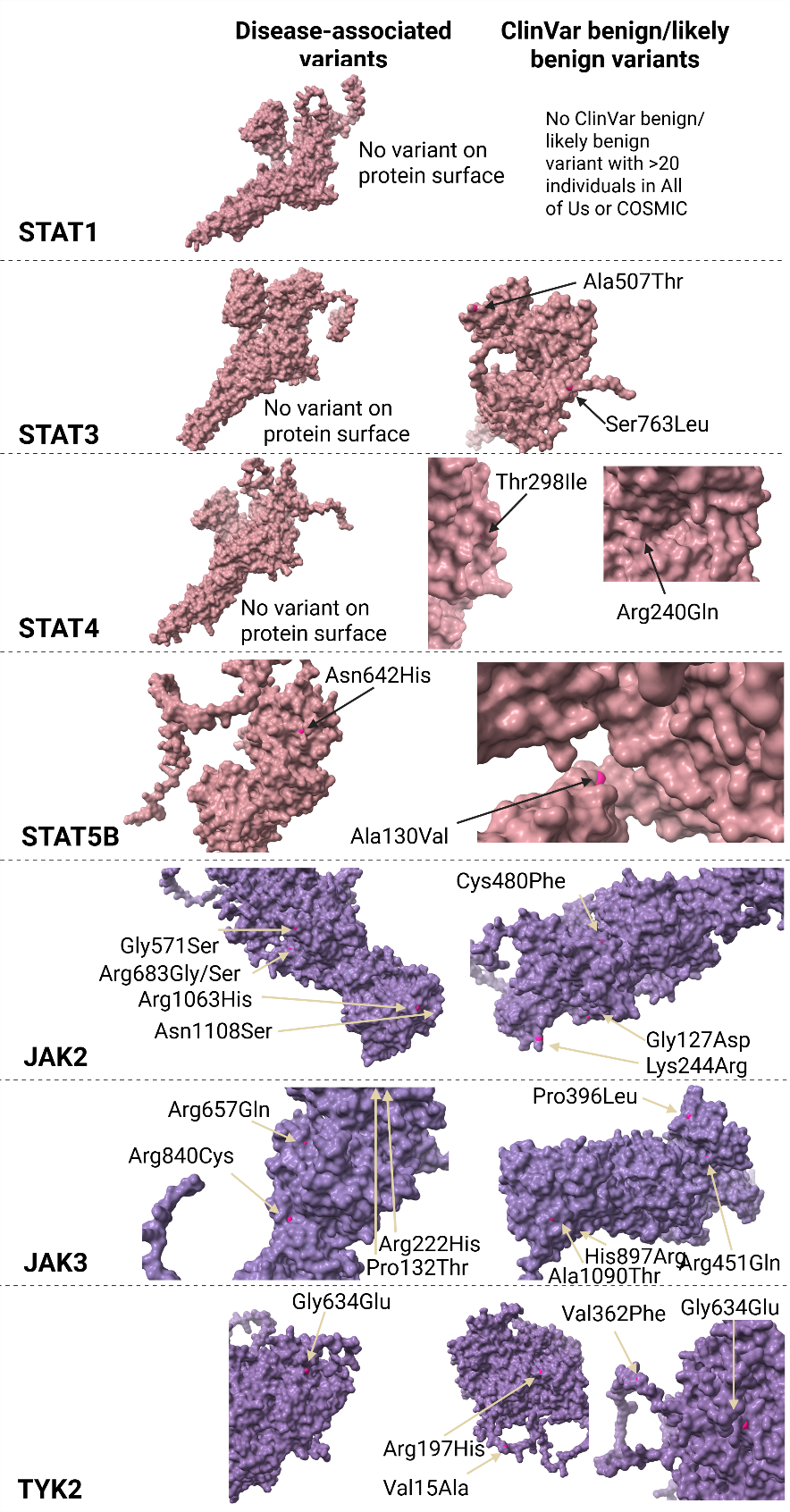


**Supplementary Figure 3:** ChimeraX visualization (AlphaFold predicted) of disease-associated and ClinVar benign/likely benign variants that face the surface of the protein.

# Supplementary Figure 4: Analysis of the sequence in 20bp proximity around disease-associated and ClinVar benign missense variant found in the All of Us and COSMIC database in terms of enzyme restriction sites


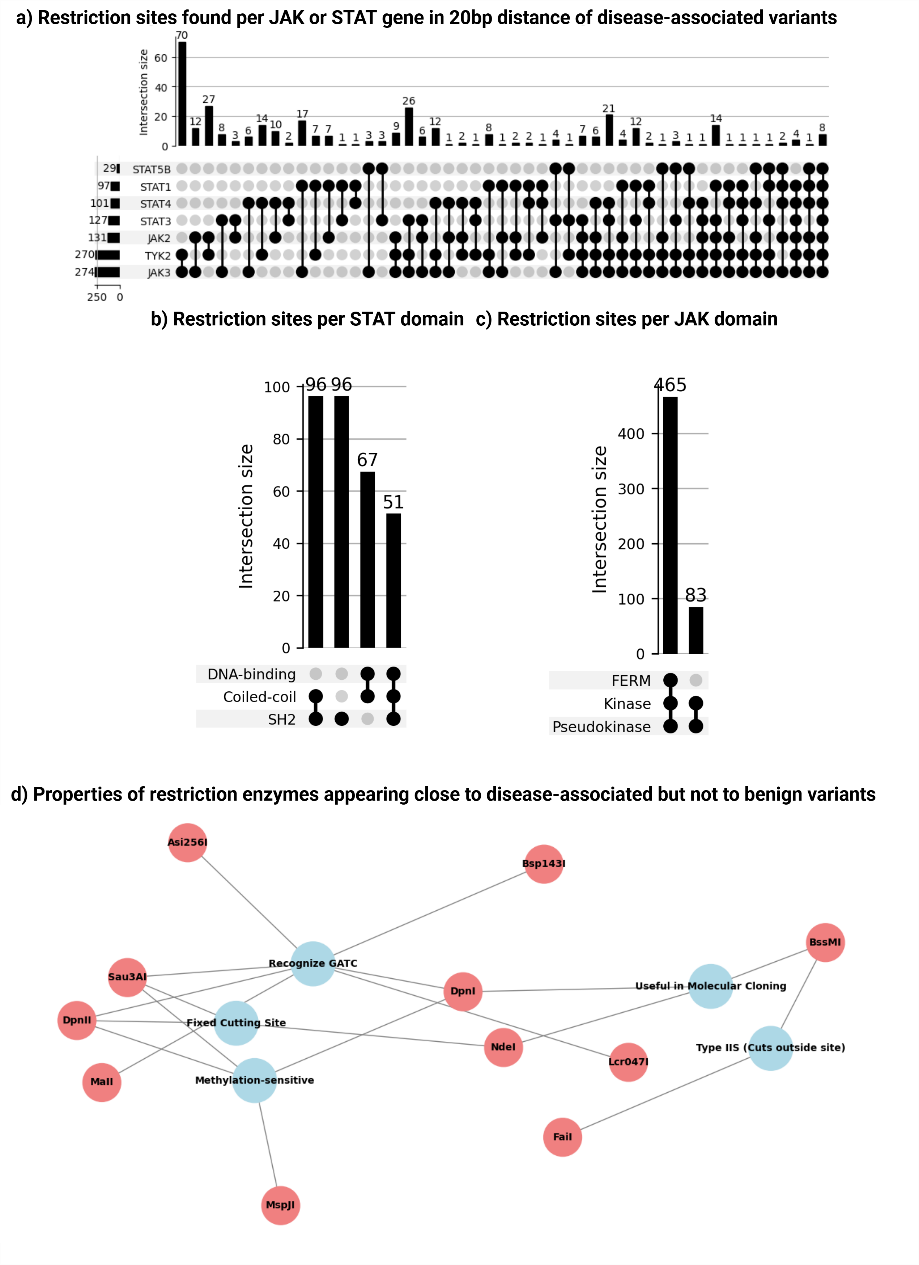


**Supplementary Figure 4:** Figures for reproducibility. Enzyme restriction site analysis in proximity to disease-associated and ClinVar benign variants in JAK and STAT genes. (a) The overlap of restriction sites found within a 20 bp distance of disease-associated mutations across different JAK and STAT genes. (b, c) Distribution of restriction sites across STAT and JAK protein domains, highlighting enrichment in DNA-binding, SH2, Pseudokinase, kinase, and FERM regions. (d) Properties of restriction enzymes found exclusively near disease-associated variants, emphasizing their functional significance in molecular cloning, methylation sensitivity, and sequence recognition.

# Supplementary Figure 5: Analysis of the sequence in 20bp proximity around disease-associated and ClinVar benign missense variant found in the All of Us and COSMIC database in terms of CRISPR cut sites


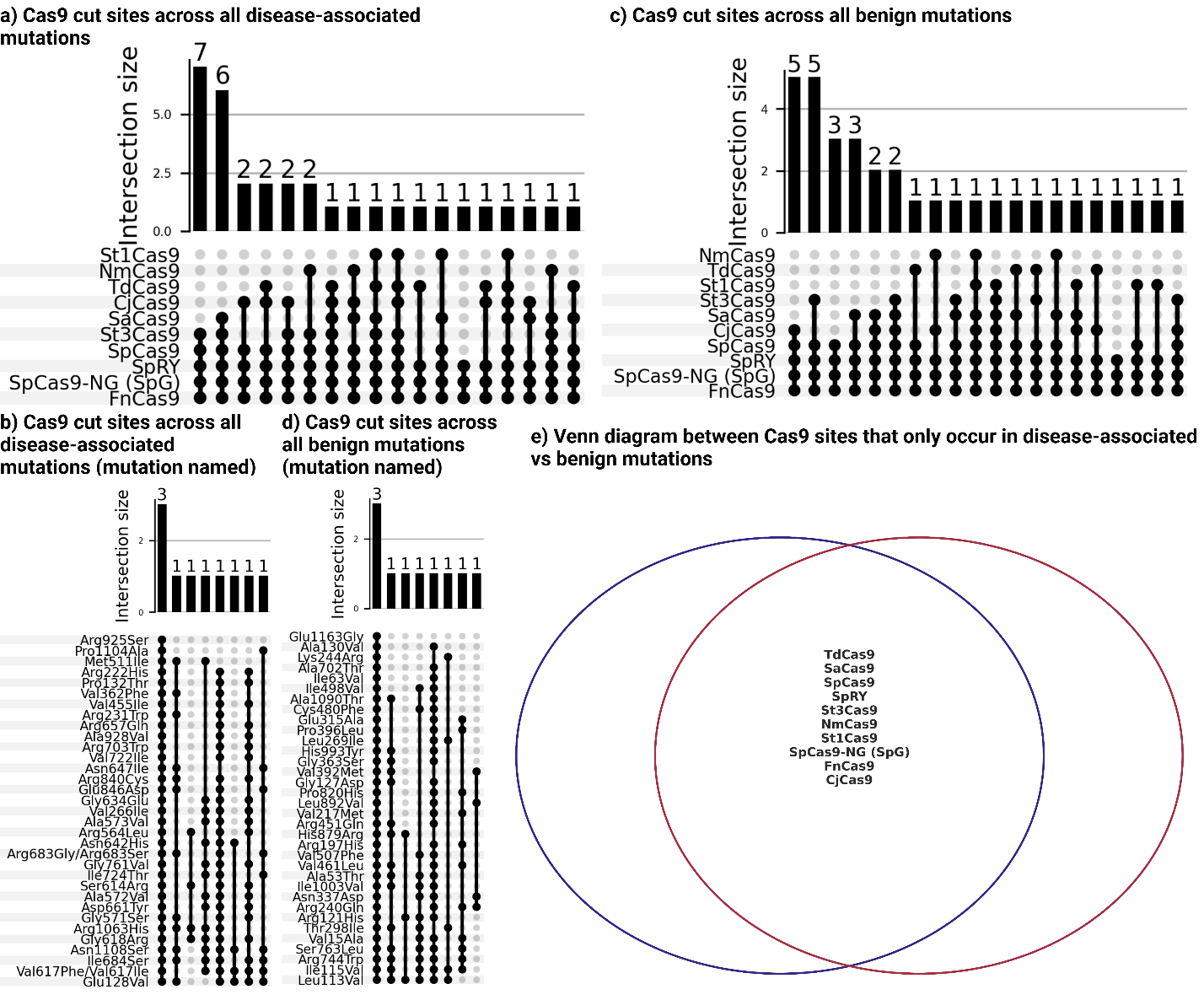


**Supplementary Figure 5:** CRISPR cut site analysis in proximity to disease-associated and benign mutations. (a) The number of Cas9 recognition sites found near disease-associated mutations across different Cas9 variants. (b, d) Distribution of Cas9 cut sites for individual disease-associated and benign mutations, respectively. (c) The number of Cas9 recognition sites found near benign mutations, providing a comparative view against pathogenic variants. (e) Venn diagram illustrating the overlap of Cas9 cut sites uniquely occurring in either disease-associated (red) or benign (blue) mutations.

# Supplementary Table 1: Amino acid changing SNPs in the STAT gene family

**NOTE: Bold written is the parent level disease-class according to Athena – OHDSI Vocabularies Repository** [**https://athena.ohdsi.org/**](https://athena.ohdsi.org/)

1. **STAT family**

| Domain | STAT1 | | STAT2 | | STAT3 | | STAT4 | | STAT5A | | STAT5B | | STAT6 | |
| --- | --- | --- | --- | --- | --- | --- | --- | --- | --- | --- | --- | --- | --- | --- |
|  | AA change  (samples) | Disease | AA change  (samples) | Disease | AA change  (samples) | Disease | AA change  (samples) | Disease | AA change  (samples) | Disease | AA change  (samples) | Disease | AA change  (samples) | Disease |
| N-terminal |  |  |  |  |  |  |  |  |  |  |  |  |  |  |
| Coiled-coil | Val266Ile (1112) | Polyendo-  crinopathy [9]  **Autoimmune disease** |  |  |  |  | Glu128Val (611) | Rheumatoid arthritis [10]  **Autoimmune disease** |  |  |  |  |  |  |
| DNA binding | Val455Ile | Chronic mucocutaneous candidiasis [11]  **Infectious disease** |  |  |  |  | Thr446Ile  (318) | Classic Kaposi Sarcoma [12]  **Cancer/ Tumor** |  |  |  |  |  |  |
| Linker |  |  |  |  | Val507Phe (27) | In silicio analysis showed that V507 is one of the most deleterious SNPs in STAT3 [13]  **No disease** |  |  |  |  |  |  |  |  |
| SH2 |  |  |  |  | Ser614Arg (COSMIC: 50) | Mixed leukemic stage [14]  **Cancer/Tumor** |  |  |  |  | Asn642His (COSMIC: 120) | Chronic myeloid neoplasms with eosinophilia and/or basophilia [15];  **Cancer/Tumor**  T-Cell Prolymphocytic Leukemia [16];  **Cancer/Tumor**  Mycobacterial osteomyelitis [17];  **Infectious disease**  Feline Alimentary T Cell Lymphoma [18]  **Cancer/Tumor** |  |  |
|  |  |  |  |  | Gly618Arg (COSMIC: 26) | Lymphocyte leukemia [19];  **Cancer/Tumor**  EBV-positive inflammatory follicular dendritic cell sarcoma [20];  **Cancer/Tumor**  T-cell large granular lymphocytic leukemia [21]  **Cancer/Tumor** |  |  |  |  |  |  |  |  |
|  |  |  |  |  | Asn647Ile (COSMIC: 30) | Large granular lymphocytic leukemia [22],  **Cancer/Tumor**  [21];  T-LGL leukemia [23], [24]  **Cancer/Tumor** |  |  |  |  |  |  |  |  |
|  |  |  |  |  | Asp661Tyr (COSMIC: 127) | T-large granular lymphocyte leukemia [24]; Large granular lymphocyte leukemia [25];  **Cancer/Tumor**  T-Cell Large Granular Lymphocytic Leukemia and Chronic Lymphoproliferative Disorder [26];  **Cancer/Tumor**  T-LGL leukemia [23];  **Cancer/Tumor**  Large granular lymphocyte leukemia [27]  **Cancer/Tumor** |  |  |  |  |  |  |  |  |
| TAD |  |  |  |  |  |  |  |  |  |  |  |  |  |  |

1. **JAK family**

| Domain | JAK2 | | JAK3 | | TYK2 | |
| --- | --- | --- | --- | --- | --- | --- |
|  | AA change  (samples) | Disease | AA change  (samples) | Disease | AA change  (samples) | Disease |
| FERM |  |  | Pro132Thr (10507) | Acute megakaryoblastic leukemia [28] [29];  **Cancer/Tumor**  Head and Neck Cancer [30];  **Cancer/Tumor**  Ameloblastoma [31]  **Cancer/Tumor** | Arg231Trp (22) | Rheumatoid arthritis [32];  **Autoimmune disease**  T-cell lymphopenia [33]  **Cancer/Tumor** |
|  |  |  | Arg222His (26) | Chronic active Epstein-Barr virus infection [34]  **Infectious disease** | Val362Phe (100467) | Tuberculosis [35];  **Infectious disease**  Systemic sclerosis [36]  **Autoimmune disease** |
| SH2 |  |  |  |  |  |  |
| SH2-linker |  |  | Met511Ile  (COSMIC: 92) | Leukemia [37] [38];  **Cancer/Tumor**  Primary T-cell Lymphomas [39]  **Cancer/Tumor** |  |  |
| Pseudokinase | Arg564Leu (54) | Myelodysplastic Syndromes [40]  **Blood disorder/Hematopoietic system** | Ala572Val (28) | T-cell malignancies [41];  JAK3-mutation-positive leukemia [42]  **Cancer/Tumor** | Gly634Glu (31) | Mycobacterial disease [43]  **Infectious disease** |
|  | Gly571Ser (289) | Acute lymphoblastic leukemia [44];  **Cancer/Tumor**  Thrombocythemia [45];  **Blood disorder/Hematopoietic system**  Myeloproliferative neoplasms [46]  **Cancer/Tumor** |  |  |  |  |
|  | His587Asn (59) | Comparative study [47]  **No disease association** | Ala573Val (28) | Cell lymphoma [48] [49] [50]  **Cancer/Tumor** | Ile684Ser (28889) | Tuberculosis [35];  **Infectious disease**  Systemic sclerosis [36];  **Autoimmune disease**  Autoimmune diseases [51];  Psoriasis-protective [52];  **Protective against autoimmune disease**  Protect against rheumatoid arthritis and autoimmunity [53]  **Protective against autoimmune disease** |
|  | Val617Phe  (All of Us: 278 COSMIC: 48,389) | Myeloproliferative neoplasms [54];  **Cancer/Tumor**  Polycythemia Vera [55];  **Blood disorder/Hematopoietic system**  Chronic Thromboembolic Pulmonary Hypertension [56];  **Blood disorder/Hematopoietic system**  Myeloproliferative neoplasms [57] [58] [59] [60] [61];  **Cancer/Tumor**  L-positive myelofibrosis [62];  **Cancer/Tumor**  Myeloid Sarcoma of the Breast [63]  **Cancer/Tumor** |  |  |  |  |
|  | Val617Ile  (COSMIC: 89) | Does not result In myeloproliferative disorder [64] | Arg657Gln  (COSMIC: 48) | Leukemia [65] [66];  **Cancer/Tumor**  Down syndrome [66]  **Genetic disorder** | Arg703Trp (2183) | Protect against rheumatoid arthritis [32]  **Protective against autoimmune disease** |
|  | Arg683Gly (COSMIC: 104)  Arg683Ser (COSMIC: 48) | Leukemia [67] [68] [69] [70] [71] [72] [73];  **Cancer/Tumor**  Netherton Syndrome [67];  **Skin disorder**  Thrombocythemia [74];  **Blood disorder/Hematopoietic system**  Lymphoma [75];  **Cancer/Tumor**  Myelodysplastic/ Myeloproliferative [71]  **Blood disorder/Hematopoietic system AND**  **Cancer/Tumor** |  |  |  |  |
|  | Ile724Thr (80) | Myeloproliferative neoplasms  [76]  **Cancer/Tumor** | Val722Ile  (All Of Us 4449, COSMIC 65) | Soft tissue sarcoma [77] [78];  **Cancer/Tumor**  Lymphoma [48];  **Cancer/Tumor**  Renal cell carcinoma [79];  **Cancer/Tumor**  HYPER FUNCTIONING PARATHYROID CYST [80];  Leukemia [81];  **Cancer/Tumor**  Idiopathic erythrocytosis [82];  **Blood disorder/Hematopoietic system** | Gly761Val (66) | Oncogenic [83];  Primary acute lymphoblastic leukemia [84]  **Cancer/Tumor** |
| Kinase | Glu846Asp (295) | Hereditary erythrocytosis with megakaryocytic atypia [85];  **Blood disorder/Hematopoietic system AND**  **Cancer/Tumor**  Erythrocytosis [86] [87]  **Blood disorder/Hematopoietic system** | Arg840Cys (93) | Cytotoxic T Lymphocyte Antigen-4-Dependent Immune Dysregulation Syndrome [88]  **Autoimmune disease** | Ala928Val (2334) | Mycobacterial disease [43];  **Infectious disease**  Systemic sclerosis [36];  **Autoimmune disease**  Protect against rheumatoid arthritis and autoimmunity [53]  **Protective against autoimmune disease** |
|  | Arg1063His (2042) | L-positive myelofibrosis [62];  **Cancer/Tumor**  Myeloproliferative neoplasms [58];  **Cancer/Tumor**  Erythrocytosis [85]  **Blood disorder/Hematopoietic system**  Ischemic Stroke [89] |  |  |  |  |
|  | Asn1108Ser (1357) | Polycythemia [90];  **Blood disorder/Hematopoietic system**  Myelofibrosis [62];  **Cancer/Tumor**  Transformation of myeloproliferative neoplasms into acute myeloid leukemia [91]  **Cancer/Tumor** | Arg925Ser (564) | Cell renal cell carcinoma [79]  **Cancer/Tumor** | Pro1104Ala (13611) | Mycobacterial disease [43];  **Infectious disease**  Autoimmune disease therapy [92];  **Protective against autoimmune disease**  Tuberculosis [93]  **Infectious disease** |

**Supplementary Table 1: Summary of missense variants in JAK and STAT proteins identified in the All of Us and COSMIC databases, organized by protein domain and disease association.** The table lists amino acid substitutions (with sample counts where available), the corresponding disease phenotype(s), and the assigned parent-level disease class used for downstream analyses.

# Supplementary Table 2: CRISPR-Cas9 PAM sequences for the different Cas9 variants.

| Name | Organism/Origin | PAM Consensus | IUPAC_PAM | References |
| --- | --- | --- | --- | --- |
| SpCas9 | Streptococcus pyogenes | 5'-NGG-3' | NGG | [94] |
| SaCas9 | Staphylococcus aureus | 5'-NNGRRT-3' | NNGRRT | [95] |
| NmCas9 | Neisseria meningitidis | 5'-NNNNGATT-3' | NNNNGATT | [96] |
| St1Cas9 | Streptococcus thermophilus CRISPR1 | 5'-NNAGAAW-3' | NNAGAAW | [97] |
| St3Cas9 | Streptococcus thermophilus CRISPR3 | 5'-NGGNG-3' | NGGNG | [98] |
| CjCas9 | Campylobacter jejuni | 5'-NNNNACA-3' | NNNNACA | [99] |
| FnCas9 | Francisella novicida | 5'-YG-3' | YG | [100] |
| TdCas9 | Treponema denticola | 5'-NAAA(A/T)-3' | NAAAW | [101] |
| xCas9 | Engineered from SpCas9 | Broad (NG, GAA, GAT, etc.) | Broad | [102] |
| SpCas9-NG (SpG) | Engineered from SpCas9 | 5'-NG-3' | NG | [103] |
| SpRY | Engineered from SpCas9 | Near-PAM-less (NRN) | NRN | [104] |
| HiFi Cas9 (HF1) | Engineered from SpCas9 | 5'-NGG-3' | NGG | [105] |
| eSpCas9 (1.1) | Engineered from SpCas9 | 5'-NGG-3' | NGG | [106] |
| HypaCas9 | Engineered from SpCas9 | 5'-NGG-3' | NGG | [107] |

# Supplementary Text 1: Limitations and considerations

While this study provides valuable insights into the distribution and surrounding properties of missense mutations in the JAK-STAT pathway that are either disease-associated or ClinVar benign, several important considerations and limitations must be acknowledged.

A key limitation is the potential dataset-specific biases and generalizability of our findings. Our analysis relies on mutations cataloged in the All of Us and COSMIC databases, which primarily represent germline and somatic mutations, respectively. While these datasets offer a broad overview, they do not comprehensively capture all population-specific variants, particularly rare mutations that could be associated with specific diseases. Additionally, the use of ClinVar for classifying benign variants introduces a potential bias, as its curation depends on submissions from various sources, some of which may be incomplete or outdated. Variants labeled as benign may still have context-dependent functional effects, such as tissue-specific impact or interactions with other genetic modifiers. Furthermore, the study focuses exclusively on missense mutations and does not account for the broader spectrum of nonsense, frameshift, or regulatory mutations, which could also contribute to JAK-STAT pathway dysregulation.

Another major limitation is the lack of functional validation for the observed mutation patterns. While we infer structural and biochemical consequences based on the location of mutations within functional domains, secondary structures, and protein surfaces, experimental verification is required to confirm these predictions. In particular, the role of a mutation is often highly dependent on cellular context—a mutation driving oncogenic transformation in one tissue type may have minimal impact on another due to differences in cellular environment, compensatory mechanisms, or regulatory feedback loops. Additionally, our structural modeling relies on AlphaFold-predicted structures, which, although highly accurate, may not fully capture conformational flexibility, post-translational modifications, or dynamic protein interactions. Experimental validation using crystallography, cryo-EM, or molecular dynamics simulations would help solidify these predictions.

Regarding the amino acid composition analysis, while we observe distinct differences in the physicochemical properties of residues near disease-associated and benign variants, these correlations do not establish causality. Certain amino acid combinations may be overrepresented due to selection biases in mutation databases rather than intrinsic mutational constraints. Moreover, the broader evolutionary conservation of these motifs and their role in protein function remains unexplored in this study. Expanding the analysis to larger and more diverse population cohorts would enhance the statistical robustness of these findings.

Beyond structural and biochemical considerations, the genomic context of mutations also presents challenges. Our study examined the presence of specific nucleotide sequences and CRISPR/Cas9 target sequences in the vicinity of disease-associated and benign mutations, yet the regulatory implications of these findings remain uncertain. The lack of strong enrichment of CRISPR cut sites near disease-associated mutations suggests that CRISPR accessibility alone does not determine mutation hotspots. However, future studies integrating chromatin accessibility, epigenetic modifications, and transcription factor binding sites could provide a deeper understanding of how these regions influence mutation susceptibility and gene regulation.

One critical limitation in the field of SNP analysis is the insufficient number of investigated SNPs due to the prolonged nature of genetic studies. Many disease-relevant mutations remain uncharacterized simply because functional studies are time- and resource-intensive. The vast majority of introduced mutations are never published [108,109], resulting in a major publication bias toward positive results. This means that the scientific community lacks access to negative experimental data, which could provide valuable information about which mutations do not cause disease and why. Increasing transparency and data-sharing practices in genetic research, including publishing negative experimental results, would greatly enhance our ability to accurately predict variant effects.

Another fundamental challenge in interpreting genetic variation is the lack of consideration for epistatic interactions between SNPs [110–112]. Current literature primarily examines single SNP effects, whereas in reality, combinations of mutations can have additive consequences on protein function, stability, cooperation, or signaling.

# REFERENCES

1. Hu X, Li J, Fu M, Zhao X, Wang W. The JAK/STAT signaling pathway: from bench to clinic. Signal Transduct Target Ther. 2021;6: 402.

2. Lee HK, Jung O, Hennighausen L. JAK inhibitors dampen activation of interferon-stimulated transcription of ACE2 isoforms in human airway epithelial cells. Commun Biol. 2021;4: 654.

3. Hoffmann M, Willruth L-L, Dietrich A, Lee HK, Knabl L, Trummer N, et al. Blood transcriptomics analysis offers insights into variant-specific immune response to SARS-CoV-2. Sci Rep. 2024;14: 1–11.

4. Banerjee S, Biehl A, Gadina M, Hasni S, Schwartz DM. JAK–STAT signaling as a target for inflammatory and autoimmune diseases: Current and future prospects. Drugs. 2017;77: 521–546.

5. Brooks AJ, Putoczki T. JAK-STAT signalling pathway in cancer. Cancers (Basel). 2020;12: 1971.

6. O’Shea JJ, Schwartz DM, Villarino AV, Gadina M, McInnes IB, Laurence A. The JAK-STAT pathway: Impact on human disease and therapeutic intervention. Annu Rev Med. 2015;66: 311–328.

7. Villarino AV, Kanno Y, O’Shea JJ. Mechanisms and consequences of Jak–STAT signaling in the immune system. Nat Immunol. 2017;18: 374–384.

8. Morris R, Kershaw NJ, Babon JJ. The molecular details of cytokine signaling via the JAK/STAT pathway. Protein Sci. 2018;27: 1984–2009.

9. Uzel G, Sampaio EP, Lawrence MG, Hsu AP, Hackett M, Dorsey MJ, et al. Dominant gain-of-function STAT1 mutations in FOXP3 wild-type immune dysregulation-polyendocrinopathy-enteropathy-X-linked-like syndrome. J Allergy Clin Immunol. 2013;131: 1611–1623.

10. Saevarsdottir S, Stefansdottir L, Sulem P, Thorleifsson G, Ferkingstad E, Rutsdottir G, et al. Multiomics analysis of rheumatoid arthritis yields sequence variants that have large effects on risk of the seropositive subset. Ann Rheum Dis. 2022;81: 1085–1095.

11. Blanco Lobo P, Lei W-T, Pelham SJ, Guisado Hernández P, Villaoslada I, de Felipe B, et al. Biallelic TRAF3IP2 variants causing chronic mucocutaneous candidiasis in a child harboring a STAT1 variant. Pediatr Allergy Immunol. 2021;32: 1804–1812.

12. Aavikko M, Kaasinen E, Nieminen JK, Byun M, Donner I, Mancuso R, et al. Whole-genome sequencing identifies*STAT4*as a putative susceptibility gene in classic Kaposi sarcoma. J Infect Dis. 2015;211: 1842–1851.

13. Ajith A, Subbiah U. In silico prediction of deleterious non-synonymous SNPs in *STAT3*. Asian Biomed (Res Rev News). 2023;17: 185–199.

14. Yan Y, Olson TL, Nyland SB, Feith DJ, Loughran TP Jr. Emergence of a STAT3 mutated NK clone in LGL leukemia. Leuk Res Rep. 2015;4: 4–7.

15. Yin CC, Tam W, Walker SM, Kaur A, Ouseph MM, Xie W, et al. *STAT5B* mutations in myeloid neoplasms differ by disease subtypes but characterize a subset of chronic myeloid neoplasms with eosinophilia and/or basophilia. Haematologica. 2023;109. doi:10.3324/haematol.2023.284311

16. Hu Z, Medeiros LJ, Xu M, Yuan J, Peker D, Shao L, et al. T-cell prolymphocytic leukemia with t(X;14)(q28;Q11.2): A clinicopathologic study of 15 cases. Am J Clin Pathol. 2023;159: 325–336.

17. Kobets AJ, Ahmad S, Boyke A, Oriko D, Holland R, Eisenberg R, et al. STAT5b gain-of-function disease in a child with mycobacterial osteomyelitis of the skull: rare presentation of an emerging disease entity. Childs Nerv Syst. 2023;39: 2071–2077.

18. Freiche V, Couronné L, Bruneau J, Hermine O. Comment on kieslinger et al. A recurrent STAT5BN642H driver mutation in feline alimentary T cell lymphoma. Cancers 2021, 13, 5238. Cancers (Basel). 2022;14: 4593.

19. Kim D, Park G, Huuhtanen J, Ghimire B, Rajala H, Moriggl R, et al. STAT3 activation in large granular lymphocyte leukemia is associated with cytokine signaling and DNA hypermethylation. Leukemia. 2021;35: 3430–3443.

20. Ramsey MC, Sabatini PJB, Watson G, Chawla T, Ko M, Sakhdari A. Case Report: Identification of a novel STAT3 mutation in EBV-positive inflammatory follicular dendritic cell sarcoma. Front Oncol. 2023;13. doi:10.3389/fonc.2023.1266897

21. Kristensen T, Larsen M, Rewes A, Frederiksen H, Thomassen M, Møller MB. Clinical relevance of sensitive and quantitative STAT3 mutation analysis using next-generation sequencing in T-cell large granular Lymphocytic leukemia. J Mol Diagn. 2014;16: 382–392.

22. Koskela HLM, Eldfors S, Ellonen P, van Adrichem AJ, Kuusanmäki H, Andersson EI, et al. Somatic*STAT3*mutations in large granular Lymphocytic leukemia. N Engl J Med. 2012;366: 1905–1913.

23. Shen M. A case report of T-LGL leukemia-associated pure red cell aplasia harboring STAT3, TNFAIP3, and KMT2D mutation. Transl Cancer Res. 2023;12: 1054–1059.

24. Cheon H, Xing JC, Moosic KB, Ung J, Chan VW, Chung DS, et al. Genomic landscape of TCRαβ and TCRγδ T-large granular lymphocyte leukemia. Blood. 2022;139: 3058–3072.

25. Olson KC, Moosic KB, Jones MK, Larkin PMK, Olson TL, Toro MF, et al. Large granular lymphocyte leukemia serum and corresponding hematological parameters reveal unique cytokine and sphingolipid biomarkers and associations with STAT3 mutations. Cancer Med. 2020;9: 6533–6549.

26. Rivero A, Mozas P, Jiménez L, López-Guerra M, Colomer D, Bataller A, et al. Clinicobiological characteristics and outcomes of patients with T-cell large granular Lymphocytic leukemia and chronic lymphoproliferative disorder of natural killer cells from a single institution. Cancers (Basel). 2021;13: 3900.

27. Tanahashi T, Sekiguchi N, Matsuda K, Takezawa Y, Ito T, Kobayashi H, et al. Cell size variations of large granular lymphocyte leukemia: Implication of a small cell subtype of granular lymphocyte leukemia with STAT3 mutations. Leuk Res. 2016;45: 8–13.

28. Riera L, Lasorsa E, Bonello L, Sismondi F, Tondat F, Di Bello C, et al. Description of a novel Janus kinase 3 P132A mutation in acute megakaryoblastic leukemia and demonstration of previously reported Janus kinase 3 mutations in normal subjects. Leuk Lymphoma. 2011;52: 1742–1750.

29. Walters DK, Mercher T, Gu T-L, O’Hare T, Tyner JW, Loriaux M, et al. Activating alleles of JAK3 in acute megakaryoblastic leukemia. Cancer Cell. 2006;10: 65–75.

30. Guerrero-Preston R, Lawson F, Rodriguez-Torres S, Noordhuis MG, Pirini F, Manuel L, et al. *JAK3* variant, immune signatures, DNA methylation, and social determinants linked to survival racial disparities in head and neck cancer patients. Cancer Prev Res (Phila). 2019;12: 255–270.

31. González-González R, López-Verdín S, Lavalle-Carrasco J, Molina-Frechero N, Isiordia-Espinoza M, Carreón-Burciaga RG, et al. Current concepts in ameloblastoma-targeted therapies in B-raf proto-oncogene serine/threonine kinase V600E mutation: Systematic review. World J Clin Oncol. 2020;11: 31–42.

32. Motegi T, Kochi Y, Matsuda K, Kubo M, Yamamoto K, Momozawa Y. Identification of rare coding variants in *TYK2* protective for rheumatoid arthritis in the Japanese population and their effects on cytokine signalling. Ann Rheum Dis. 2019;78: 1062–1069.

33. Nemoto M, Hattori H, Maeda N, Akita N, Muramatsu H, Moritani S, et al. Compound heterozygous TYK2 mutations underlie primary immunodeficiency with T-cell lymphopenia. Sci Rep. 2018;8. doi:10.1038/s41598-018-25260-8

34. Zhong L, Wang W, Ma M, Gou L, Tang X, Song H. Chronic active Epstein–Barr virus infection as the initial symptom in a Janus kinase 3 deficiency child. Medicine (Baltimore). 2017;96: e7989.

35. Kerner G, Ramirez-Alejo N, Seeleuthner Y, Yang R, Ogishi M, Cobat A, et al. Homozygosity for *TYK2* P1104A underlies tuberculosis in about 1% of patients in a cohort of European ancestry. Proc Natl Acad Sci U S A. 2019;116: 10430–10434.

36. López-Isac E, Campillo-Davo D, Bossini-Castillo L, Guerra SG, Assassi S, Simeón CP, et al. Influence of*TYK2*in systemic sclerosis susceptibility: a new*locus*in the IL-12 pathway. Ann Rheum Dis. 2016;75: 1521–1526.

37. Yuan S, Wang X, Hou S, Guo T, Lan Y, Yang S, et al. PHF6 and JAK3 mutations cooperate to drive T-cell acute lymphoblastic leukemia progression. Leukemia. 2022;36: 370–382.

38. Si H, Wang J, He R, Yu X, Li S, Huang J, et al. Identification of U937JAK3-M511I acute myeloid leukemia cells as a sensitive model to JAK3 inhibitor. Front Oncol. 2022;11. doi:10.3389/fonc.2021.807200

39. Yim J, Koh J, Kim S, Song SG, Bae JM, Yun H, et al. Clinicopathologic and genetic features of primary T-cell lymphomas of the central nervous system. Am J Surg Pathol. 2022;46: 486–497.

40. Delio M, Bryke C, Mendez L, Joseph L, Jassim S. JAK2 mutations are rare and diverse in myelodysplastic syndromes: Case series and review of the literature. Hematol Rep. 2023;15: 73–87.

41. Rivera-Munoz P, Laurent AP, Siret A, Lopez CK, Ignacimouttou C, Cornejo MG, et al. Partial trisomy 21 contributes to T-cell malignancies induced by JAK3-activating mutations in murine models. Blood Adv. 2018;2: 1616–1627.

42. Agarwal A, MacKenzie RJ, Eide CA, Davare MA, Watanabe-Smith K, Tognon CE, et al. Functional RNAi screen targeting cytokine and growth factor receptors reveals oncorequisite role for interleukin-2 gamma receptor in JAK3-mutation-positive leukemia. Oncogene. 2015;34: 2991–2999.

43. Ogishi M, Arias AA, Yang R, Han JE, Zhang P, Rinchai D, et al. Impaired IL-23–dependent induction of IFN-γ underlies mycobacterial disease in patients with inherited TYK2 deficiency. J Exp Med. 2022;219. doi:10.1084/jem.20220094

44. Lin M, Nebral K, Gertzen CGW, Ganmore I, Haas OA, Bhatia S, et al. JAK2 p.G571S in B-cell precursor acute lymphoblastic leukemia: a synergizing germline susceptibility. Leukemia. 2019;33: 2331–2335.

45. Panovska-Stavridis I, Eftimov A, Ivanovski M, Pivkova-Veljanovska A, Cevreska L, Hermouet S, et al. Essential thrombocythemia associated with germline JAK2 G571S variant and somatic CALR type 1 mutation. Clin Lymphoma Myeloma Leuk. 2016;16: e55–e57.

46. Bahar B, Barton K, Kini AR. The role of the Exon 13 G571S JAK2 mutation in myeloproliferative neoplasms. Leuk Res Rep. 2016;6: 27–28.

47. Lee T-S, Ma W, Zhang X, Kantarjian H, Albitar M. Structural effects of clinically observed mutations in JAK2 exons 13-15: comparison with V617F and exon 12 mutations. BMC Struct Biol. 2009;9: 58.

48. Bouchekioua A, Scourzic L, de Wever O, Zhang Y, Cervera P, Aline-Fardin A, et al. JAK3 deregulation by activating mutations confers invasive growth advantage in extranodal nasal-type natural killer cell lymphoma. Leukemia. 2014;28: 338–348.

49. Sim SH, Kim S, Kim TM, Jeon YK, Nam SJ, Ahn Y-O, et al. Novel JAK3-activating mutations in extranodal NK/T-cell lymphoma, nasal type. Am J Pathol. 2017;187: 980–986.

50. Koo GC, Tan SY, Tang T, Poon SL, Allen GE, Tan L, et al. Janus kinase 3–activating mutations identified in natural killer/T-cell lymphoma. Cancer Discov. 2012;2: 591–597.

51. Li Z, Gakovic M, Ragimbeau J, Eloranta M-L, Rönnblom L, Michel F, et al. Two rare disease-associated Tyk2 variants are catalytically impaired but signaling competent. J Immunol. 2013;190: 2335–2344.

52. Enerbäck C, Sandin C, Lambert S, Zawistowski M, Stuart PE, Verma D, et al. The psoriasis-protective TYK2 I684S variant impairs IL-12 stimulated pSTAT4 response in skin-homing CD4+ and CD8+ memory T-cells. Sci Rep. 2018;8. doi:10.1038/s41598-018-25282-2

53. Diogo D, Bastarache L, Liao KP, Graham RR, Fulton RS, Greenberg JD, et al. TYK2 protein-coding variants protect against rheumatoid arthritis and autoimmunity, with no evidence of major pleiotropic effects on non-autoimmune complex traits. PLoS One. 2015;10: e0122271.

54. Zhang Y, Zhao Y, Liu Y, Zhang M, Zhang J. New advances in the role of *JAK2* V617F mutation in myeloproliferative neoplasms. Cancer. 2024 [cited 16 Sep 2024]. doi:10.1002/cncr.35559

55. Haji Paiman NS, Mat Nasir N, Miptah HN, Saidon N, Abdul Monir M. Challenges in diagnosing polycythemia Vera in primary care: A 55-year-old Malaysian woman with atypical presentation. Am J Case Rep. 2024;25. doi:10.12659/ajcr.944202

56. Eichstaedt CA, Verweyen J, Halank M, Benjamin N, Fischer C, Mayer E, et al. Myeloproliferative diseases as possible risk factor for development of chronic thromboembolic pulmonary hypertension—A genetic study. Int J Mol Sci. 2020;21: 3339.

57. Bourrienne M-C, Loyau S, Faille D, Gay J, Akhenak S, Farkh C, et al. Impaired fibrinolysis in JAK2V617F-related myeloproliferative neoplasms. J Thromb Haemost. 2024 [cited 16 Sep 2024]. doi:10.1016/j.jtha.2024.07.031

58. Mambet C, Babosova O, Defour J-P, Leroy E, Necula L, Stanca O, et al. Cooccurring JAK2 V617F and R1063H mutations increase JAK2 signaling and neutrophilia in myeloproliferative neoplasms. Blood. 2018;132: 2695–2699.

59. Patchell D, Keohane C, O’Shea S, Langabeer SE. Incidence and impact of non-canonical JAK2 p.(Val617Phe) mutations in myeloproliferative neoplasm molecular diagnostics. J Clin Pathol. 2024; jcp-2023-209276.

60. Choi DC, Messali N, Uda NR, Abu-Zeinah G, Kermani P, Yabut MM, et al. JAK2V617F impairs lymphoid differentiation in myeloproliferative neoplasms. Leukemia. 2024 [cited 16 Sep 2024]. doi:10.1038/s41375-024-02388-3

61. Veitia RA, Innan H. Pathogenic “germline” variants associated with myeloproliferative disorders in apparently normal individuals: Inherited or acquired genetic alterations? Clin Genet. 2022;101: 371–374.

62. Schulze S, Stengel R, Jaekel N, Wang S-Y, Franke G-N, Roskos M, et al. Concomitant and noncanonical *JAK2* and *MPL* mutations in *JAK2*V617F‐ and *MPLW*515 L‐positive myelofibrosis. Genes Chromosomes Cancer. 2019;58: 747–755.

63. Pace M, Guadagno E, Russo D, Gencarelli A, Carlea A, Di Spiezio A, et al. Myeloid sarcoma of the breast as blast phase of JAK2-mutated (Val617Phe Exon 14p) essential thrombocythemia: A case report and a systematic literature review. Pathobiology. 2023;90: 123–130.

64. Brooks SA, Luty SB, Lai HY, Morse SJ, Nguyen TK, Royer LR, et al. JAK2V617I results in cytokine hypersensitivity without causing an overt myeloproliferative disorder in a mouse transduction–transplantation model. Exp Hematol. 2016;44: 24-29.e1.

65. Bergmann AK, Schneppenheim S, Seifert M, Betts MJ, Haake A, Lopez C, et al. Recurrent mutation of *JAK3* in T‐cell prolymphocytic leukemia. Genes Chromosomes Cancer. 2014;53: 309–316.

66. Sato T, Toki T, Kanezaki R, Xu G, Terui K, Kanegane H, et al. Functional analysis of *JAK3* mutations in transient myeloproliferative disorder and acute megakaryoblastic leukaemia accompanying Down syndrome. Br J Haematol. 2008;141: 681–688.

67. Skoczen S, Stepien K, Mlynarski W, Centkowski P, Kwiecinska K, Korostynski M, et al. Genetic signature of acute lymphoblastic leukemia and netherton syndrome co-incidence—first report in the literature. Front Oncol. 2020;9. doi:10.3389/fonc.2019.01477

68. Hassan NM, Abdellateif MS, Radwan EM, Hameed SA, Desouky EDE, Kamel MM, et al. Prognostic significance of CRLF2 overexpression and JAK2 mutation in Egyptian pediatric patients with B-precursor acute lymphoblastic leukemia. Clin Lymphoma Myeloma Leuk. 2022;22: e376–e385.

69. Carreño-Tarragona G, Varghese LN, Sebastián E, Gálvez E, Marín-Sánchez A, López-Muñoz N, et al. A typical acute lymphoblastic leukemia JAK2 variant, R683G, causes an aggressive form of familial thrombocytosis when germline. Leukemia. 2021;35: 3295–3298.

70. Gupta DG, Varma N, Sreedharanunni S, Abdulkadir SA, Naseem S, Sachdeva MUS, et al. ‘Evaluation of adverse prognostic gene alterations & MRD positivity in BCR::ABL1-like B-lineage acute lymphoblastic leukaemia patients, in a resource-constrained setting. Br J Cancer. 2023;129: 143–152.

71. Krah NM, Miotke L, Li P, Patel JL, Bowen AR, Pomicter AD, et al. JAK2 R683S mutation resulting in dual diagnoses of chronic eosinophilic leukemia and myelodysplastic/myeloproliferative overlap syndrome. J Natl Compr Canc Netw. 2023;21: 1218–1223.

72. Gupta DG, Varma N, Kumar A, Naseem S, Sachdeva MUS, Sreedharanunni S, et al. Genomic and proteomic characterization of Philadelphia‐like B‐lineage acute lymphoblastic leukemia: A report of Indian patients. Cancer. 2023;129: 1217–1226.

73. Xu RZ, Karsan A, Xu Z, Berry BR. A rare de novo pure erythroid leukemia with JAK2 R683S mutation. Ann Hematol. 2022;101: 921–922.

74. Arai A, Yoshimitsu M, Otsuka M, Ito Y, Miyazono T, Nakano N, et al. Identification of putative noncanonical driver mutations in patients with essential thrombocythemia. Eur J Haematol. 2023;110: 639–647.

75. Roncero AM, López-Nieva P, Cobos-Fernández MA, Villa-Morales M, González-Sánchez L, López-Lorenzo JL, et al. Contribution of JAK2 mutations to T-cell lymphoblastic lymphoma development. Leukemia. 2016;30: 94–103.

76. Puli’uvea C, Immanuel T, Green TN, Tsai P, Shepherd PR, Kalev-Zylinska ML. Insights into the role of JAK2-I724T variant in myeloproliferative neoplasms from a unique cohort of New Zealand patients. Hematology. 2024;29. doi:10.1080/16078454.2023.2297597

77. Xu L, Wilson RA, Laetsch TW, Oliver D, Spunt SL, Hawkins DS, et al. Potential pitfalls of mass spectrometry to uncover mutations in childhood soft tissue sarcoma: A report from the Children’s Oncology Group. Sci Rep. 2016;6. doi:10.1038/srep33429

78. Ehrentraut S, Schneider B, Nagel S, Pommerenke C, Quentmeier H, Geffers R, et al. Th17 cytokine differentiation and loss of plasticity after SOCS1 inactivation in a cutaneous T-cell lymphoma. Oncotarget. 2016;7: 34201–34216.

79. de Martino M, Gigante M, Cormio L, Prattichizzo C, Cavalcanti E, Gigante M, et al. JAK3 in clear cell renal cell carcinoma: Mutational screening and clinical implications. Urol Oncol. 2013;31: 930–937.

80. Alghamdi K. Delayed diagnosis of a hyper functioning parathyroid cyst. A case report and genetic analysis. Acta Endocrinol (Buchar). 2016;12: 215–218.

81. Yin C, Sandoval C, Baeg G-H. Identification of mutant alleles of*JAK3*in pediatric patients with acute lymphoblastic leukemia. Leuk Lymphoma. 2015;56: 1502–1506.

82. Elli EM, Mauri M, D’Aliberti D, Crespiatico I, Fontana D, Redaelli S, et al. Idiopathic erythrocytosis: a germline disease? Clin Exp Med. 2024;24. doi:10.1007/s10238-023-01283-y

83. Woess K, Macho-Maschler S, Van Ingen Schenau DS, Butler M, Lassnig C, Valcanover D, et al. Oncogenic TYK2 P760L kinase is effectively targeted by combinatorial TYK2, mTOR and CDK4/6 kinase blockade. Haematologica. 2022;108: 993–1005.

84. Waanders E, Scheijen B, Jongmans MCJ, Venselaar H, van Reijmersdal SV, van Dijk AHA, et al. Germline activating TYK2 mutations in pediatric patients with two primary acute lymphoblastic leukemia occurrences. Leukemia. 2017;31: 821–828.

85. Kapralova K, Horvathova M, Pecquet C, Fialova Kucerova J, Pospisilova D, Leroy E, et al. Cooperation of germ line JAK2 mutations E846D and R1063H in hereditary erythrocytosis with megakaryocytic atypia. Blood. 2016;128: 1418–1423.

86. Tun PWW, Buka RJ, Graham J, Dyer P. Heterozygous, germline *JAK2* E846D substitution as the cause of familial erythrocytosis. Br J Haematol. 2022;198: 923–926.

87. Maaziz N, Garrec C, Airaud F, Bobée V, Contentin N, Cayssials E, et al. Germline JAK2 E846D substitution as the cause of erythrocytosis? Genes (Basel). 2023;14: 1066.

88. Sic H, Speletas M, Cornacchione V, Seidl M, Beibel M, Linghu B, et al. An activating Janus kinase-3 mutation is associated with cytotoxic T lymphocyte antigen-4-dependent immune dysregulation syndrome. Front Immunol. 2017;8. doi:10.3389/fimmu.2017.01824

89. Ilinca A, Martinez-Majander N, Samuelsson S, Piccinelli P, Truvé K, Cole J, et al. Whole-exome sequencing in 22 young ischemic stroke patients with familial clustering of stroke. Stroke. 2020;51: 1056–1063.

90. Oliveira e Costa A, Barreira A, Cunha M, Salvador F. Polycythemia and JAK2 variant N1108S: cause-and-effect or coincidence? Hematol Transfus Cell Ther. 2023. doi:10.1016/j.htct.2023.01.006

91. Benton CB, Boddu PC, DiNardo CD, Bose P, Wang F, Assi R, et al. Janus kinase 2 variants associated with the transformation of myeloproliferative neoplasms into acute myeloid leukemia. Cancer. 2019;125: 1855–1866.

92. Jensen LT, Attfield KE, Feldmann M, Fugger L. Allosteric TYK2 inhibition: redefining autoimmune disease therapy beyond JAK1-3 inhibitors. EBioMedicine. 2023;97: 104840.

93. Kerner G, Laval G, Patin E, Boisson-Dupuis S, Abel L, Casanova J-L, et al. Human ancient DNA analyses reveal the high burden of tuberculosis in Europeans over the last 2,000 years. Am J Hum Genet. 2021;108: 517–524.

94. Guo M, Ren K, Zhu Y, Tang Z, Wang Y, Zhang B, et al. Structural insights into a high fidelity variant of SpCas9. Cell Res. 2019;29: 183–192.

95. Du W, Zhu H, Qian J, Xue D, Zheng S, Huang Q. Full-length model of SaCas9-sgRNA-DNA complex in cleavage state. Int J Mol Sci. 2023;24: 1204.

96. Hou Z, Zhang Y, Propson NE, Howden SE, Chu L-F, Sontheimer EJ, et al. Efficient genome engineering in human pluripotent stem cells using Cas9 from Neisseria meningitidis. Proc Natl Acad Sci U S A. 2013;110: 15644–15649.

97. Zhang Y, Zhang H, Xu X, Wang Y, Chen W, Wang Y, et al. Catalytic-state structure and engineering of Streptococcus thermophilus Cas9. Nat Catal. 2020;3: 813–823.

98. Müller M, Lee CM, Gasiunas G, Davis TH, Cradick TJ, Siksnys V, et al. Streptococcus thermophilus CRISPR-Cas9 systems enable specific editing of the human genome. Mol Ther. 2016;24: 636–644.

99. Schmidheini L, Mathis N, Marquart KF, Rothgangl T, Kissling L, Böck D, et al. Continuous directed evolution of a compact CjCas9 variant with broad PAM compatibility. Nat Chem Biol. 2024;20: 333–343.

100. Acharya S, Ansari AH, Kumar Das P, Hirano S, Aich M, Rauthan R, et al. PAM-flexible Engineered FnCas9 variants for robust and ultra-precise genome editing and diagnostics. Nat Commun. 2024;15: 5471.

101. Wu J, Tang B, Tang Y. Allele-specific genome targeting in the development of precision medicine. Theranostics. 2020;10: 3118–3137.

102. Kim HK, Lee S, Kim Y, Park J, Min S, Choi JW, et al. High-throughput analysis of the activities of xCas9, SpCas9-NG and SpCas9 at matched and mismatched target sequences in human cells. Nat Biomed Eng. 2020;4: 111–124.

103. Liang F, Zhang Y, Li L, Yang Y, Fei J-F, Liu Y, et al. SpG and SpRY variants expand the CRISPR toolbox for genome editing in zebrafish. Nat Commun. 2022;13: 1–10.

104. Hibshman GN, Bravo JPK, Hooper MM, Dangerfield TL, Zhang H, Finkelstein IJ, et al. Unraveling the mechanisms of PAMless DNA interrogation by SpRY-Cas9. Nat Commun. 2024;15: 1–15.

105. Vakulskas CA, Dever DP, Rettig GR, Turk R, Jacobi AM, Collingwood MA, et al. A high-fidelity Cas9 mutant delivered as a ribonucleoprotein complex enables efficient gene editing in human hematopoietic stem and progenitor cells. Nat Med. 2018;24: 1216–1224.

106. Slaymaker IM, Gao L, Zetsche B, Scott DA, Yan WX, Zhang F. Rationally engineered Cas9 nucleases with improved specificity. Science. 2016;351: 84–88.

107. Ikeda A, Fujii W, Sugiura K, Naito K. High-fidelity endonuclease variant HypaCas9 facilitates accurate allele-specific gene modification in mouse zygotes. Commun Biol. 2019;2: 371.

108. Hoffmann M, Vaz T, Chhatrala S, Hennighausen L. Data-driven projections of candidate enhancer-activating SNPs in immune regulation. BMC Genomics. 2025;26. doi:10.1186/s12864-025-11374-7

109. Hoffmann M, Hennighausen L. Spotlight on amino acid changing mutations in the JAK-STAT pathway: from disease-specific mutation to general mutation databases. Sci Rep. 2025;15: 1–12.

110. Hoffmann M, Poschenrieder JM, Incudini M, Baier S, Fritz A, Maier A, et al. Network medicine-based epistasis detection in complex diseases: ready for quantum computing. Nucleic Acids Res. 2024;52: 10144–10160.

111. Hernández-Lorenzo L, Hoffmann M, Scheibling E, List M, Matías-Guiu JA, Ayala JL. On the limits of graph neural networks for the early diagnosis of Alzheimer’s disease. Sci Rep. 2022;12: 17632.

112. Blumenthal DB, Baumbach J, Hoffmann M, Kacprowski T, List M. A framework for modeling epistatic interaction. Bioinformatics. 2020. doi:10.1093/bioinformatics/btaa990
