# Supplementary material for: How Genomic and Structural Context Could Shape JAK-STAT Variant Pathogenicity
Source: Twin Res Hum Genet. 2026 Mar 31:1–13. Online ahead of print. doi: 10.1017/thg.2026.10054 (PMC13107192; doi:10.1017/thg.2026.10054)
Supplement: Hoffmann and Lee supplementary material 2 — Hoffmann and Lee supplementary material [file S1832427426100541sup002.docx]

*Supplementary Materials (cDNA and amino acid positions) for:* **How genomic and structural context could shape JAK-STAT variant pathogenicity and benignity**

Markus Hoffmann^1,*^ and Hye Kyung Lee^1^

^1^ National Institute of Diabetes, Digestive, and Kidney Diseases, Bethesda, MD 20892, United States of America

*corresponding authors: [markus.hoffmann@nih.gov](mailto:markus.hoffmann@nih.gov); [hyekyung.lee@nih.gov](mailto:hyekyung.lee@nih.gov)

**ABSTRACT**

The Janus kinase (JAK)-Signal Transducer and Activator of Transcription (STAT) pathway is essential for cellular signal transduction, regulating immune responses, hematopoiesis, and cell proliferation. Dysregulation of JAK-STAT signaling due to genetic variations, particularly missense mutations, has been implicated in autoimmune disorders, cancers, and hematological malignancies. This study investigates missense mutations in JAK and STAT genes, focusing on disease-associated single nucleotide polymorphisms (SNPs) and ClinVar benign variants identified in the All of Us and COSMIC databases. We analyzed the distribution of these mutations across functional domains, their structural localization, and biochemical properties. We identified mutation hotspots within specific domains, highlighting their correlation with disease phenotypes. Structural mapping revealed that disease-associated SNPs predominantly localize in linker regions and at the boundaries of secondary structures, suggesting a more significant impact on protein stability and function of the JAK and STAT gene families. Additionally, we examined the genomic context of mutations and identified distinct patterns in enzyme restriction sites, particularly the “GATC” nucleotide sequence. Furthermore, our analysis found no predominant association between CRISPR-Cas9 target sites and ClinVar benign/disease-associated SNPs. Analysis of amino acid sequence patterns surrounding mutations uncovered unique patterns distinguishing disease-associated from benign variants, offering insights into mutation susceptibility. Our findings emphasize the importance of structural and biochemical context in determining pathogenicity. With this study, we provide a foundation for refining variant classification and understanding JAK-STAT pathway mutations in disease.

**Keywords:** JAK-STAT pathway, missense mutations, SNPs, protein structure, disease-associated variants, COSMIC, All of Us database, ClinVar

Contents

[STAT1 2](#_Toc189745765)

[Amino Acid Domain positions to Nucleotide positions 2](#_Toc189745766)

[Mutation positions to Nucleotide positions 2](#_Toc189745767)

[STAT3 3](#_Toc189745768)

[Amino Acid Domain positions to Nucleotide positions 3](#_Toc189745769)

[Mutation positions to Nucleotide positions 3](#_Toc189745770)

[STAT4 4](#_Toc189745771)

[Amino Acid Domain positions to Nucleotide positions 4](#_Toc189745772)

[Mutation positions to Nucleotide positions 4](#_Toc189745773)

[STAT5B 5](#_Toc189745774)

[Amino Acid Domain positions to Nucleotide positions 5](#_Toc189745775)

[Mutation positions to Nucleotide positions 5](#_Toc189745776)

[JAK2 6](#_Toc189745777)

[Amino Acid Domain positions to Nucleotide positions 6](#_Toc189745778)

[Mutation positions to Nucleotide positions 6](#_Toc189745779)

[JAK3 7](#_Toc189745780)

[Amino Acid Domain positions to Nucleotide positions 7](#_Toc189745781)

[Mutation positions to Nucleotide positions 7](#_Toc189745782)

[TYK2 8](#_Toc189745783)

[Amino Acid Domain positions to Nucleotide positions 8](#_Toc189745784)

[Mutation positions to Nucleotide positions 8](#_Toc189745785)

**Genome version is HG38!**

# STAT1

## Amino Acid Domain positions to Nucleotide positions

| Domain | Start AA | End AA | Start Nucleotide | End Nucleotide |
| --- | --- | --- | --- | --- |
| N-terminal | 1 | 121 | 3 | 363 |
| Coiled-coil | 122 | 313 | 364 | 939 |
| DNA-binding | 314 | 477 | 940 | 1431 |
| Linker | 478 | 558 | 1432 | 1674 |
| SH2 | 559 | 707 | 1675 | 2121 |
| TAD | 708 | 750 | 2122 | 2250 |

## Mutation positions to Nucleotide positions

| Mutation | ACTUAL Start Nucleotide | ACTUAL End Nucleotide |
| --- | --- | --- |
| Val266Ile | 796 | 798 |
| Val455Ile | 1363 | 1365 |

# STAT3

## Amino Acid Domain positions to Nucleotide positions

| Domain | Start AA | End AA | Start Nucleotide | End Nucleotide |
| --- | --- | --- | --- | --- |
| N-terminal | 1 | 122 | 3 | 366 |
| Coiled-coil | 123 | 318 | 367 | 954 |
| DNA-binding | 319 | 484 | 955 | 1452 |
| Linker | 485 | 565 | 1453 | 1695 |
| SH2 | 566 | 716 | 1696 | 2148 |
| TAD | 717 | 770 | 2149 | 2310 |

## Mutation positions to Nucleotide positions

| Mutation | Start Nucleotide | End Nucleotide |
| --- | --- | --- |
| Ser614Arg | 1840 | 1842 |
| Gly618Arg | 1852 | 1854 |
| Asn647Ile | 1939 | 1941 |
| Asp661Tyr | 1981 | 1983 |

STAT4

Amino Acid Domain positions to Nucleotide positions

| Domain | Start AA | End AA | Start Nucleotide | End Nucleotide |
| --- | --- | --- | --- | --- |
| N-terminal | 1 | 122 | 3 | 366 |
| Coiled-coil | 123 | 312 | 367 | 936 |
| DNA-binding | 313 | 473 | 937 | 1419 |
| Linker | 474 | 554 | 1420 | 1662 |
| SH2 | 555 | 700 | 1663 | 2100 |
| TAD | 701 | 748 | 2101 | 2244 |

Mutation positions to Nucleotide positions

| Mutation | Start Nucleotide | End Nucleotide |
| --- | --- | --- |
| Glu128Val | 382 | 384 |
| Thr446Ile | 1336 | 1338 |

# STAT5B

## Amino Acid Domain positions to Nucleotide positions

| Domain | Start AA | End AA | Start Nucleotide | End Nucleotide |
| --- | --- | --- | --- | --- |
| N-terminal | 1 | 125 | 3 | 375 |
| Coiled-coil | 126 | 330 | 376 | 990 |
| DNA-binding | 331 | 489 | 991 | 1467 |
| Linker | 490 | 574 | 1468 | 1722 |
| SH2 | 575 | 712 | 1723 | 2136 |
| TAD | 713 | 794 | 2137 | 2358 |

## Mutation positions to Nucleotide positions

| Mutation | Start Nucleotide | End Nucleotide |
| --- | --- | --- |
| Asn642His | 1924 | 1926 |

# JAK2

## Amino Acid Domain positions to Nucleotide positions

| Domain | Start AA | End AA | Start Nucleotide | End Nucleotide |
| --- | --- | --- | --- | --- |
| FERM | 37 | 380 | 111 | 1140 |
| SH2 | 386 | 482 | 1158 | 1446 |
| Pseudokinase | 545 | 809 | 1635 | 2427 |
| Kinase | 844 | 1132 | 2532 | 3396 |

## Mutation positions to Nucleotide positions

| Mutation | Start Nucleotide | End Nucleotide |
| --- | --- | --- |
| Arg564Leu | 1690 | 1692 |
| Gly571Ser | 1711 | 1713 |
| Val617Phe/Val617Ile | 1849 | 1851 |
| Arg683Gly/Arg683Ser | 2047 | 2049 |
| Ile724Thr | 2170 | 2172 |
| Glu846Asp | 2536 | 2538 |
| Arg1063His | 3187 | 3189 |
| Asn1108Ser | 3322 | 3324 |

# JAK3

## Amino Acid Domain positions to Nucleotide positions

| Domain | Start AA | End AA | Start Nucleotide | End Nucleotide |
| --- | --- | --- | --- | --- |
| FERM | 24 | 356 | 72 | 1068 |
| SH2 | 362 | 458 | 1086 | 1374 |
| Pseudokinase | 521 | 781 | 1563 | 2343 |
| Kinase | 822 | 1124 | 2466 | 3372 |

## Mutation positions to Nucleotide positions

| Mutation | Start Nucleotide | End Nucleotide |
| --- | --- | --- |
| Pro132Thr | 394 | 396 |
| Arg222His | 664 | 666 |
| Met511Ile | 1531 | 1533 |
| Ala572Val | 1714 | 1716 |
| Ala573Val | 1717 | 1719 |
| Arg657Gln | 1969 | 1971 |
| Val722Ile | 2164 | 2166 |
| Arg840Cys | 2518 | 2520 |
| Arg925Ser | 2773 | 2775 |

# TYK2

## Amino Acid Domain positions to Nucleotide positions

| Domain | Start AA | End AA | Start Nucleotide | End Nucleotide |
| --- | --- | --- | --- | --- |
| FERM | 26 | 431 | 78 | 1293 |
| SH2 | 452 | 539 | 1356 | 1617 |
| Pseudokinase | 589 | 875 | 1767 | 2625 |
| Kinase | 892 | 1187 | 2676 | 3561 |

## Mutation positions to Nucleotide positions

| Mutation | Start Nucleotide | End Nucleotide |
| --- | --- | --- |
| Arg231Trp | 691 | 693 |
| Val362Phe | 1084 | 1086 |
| Gly634Glu | 1900 | 1902 |
| Ile684Ser | 2050 | 2052 |
| Arg703Trp | 2107 | 2109 |
| Gly761Val | 2281 | 2283 |
| Ala928Val | 2782 | 2784 |
| Pro1104Ala | 3310 | 3312 |
